# Microscopic Theory of Polaron-Polariton Dispersion and Propagation

**DOI:** 10.1021/acs.nanolett.5c04134

**Published:** 2025-10-20

**Authors:** Logan Blackham, Arshath Manjalingal, Saeed Rahmanian Koshkaki, Arkajit Mandal

**Affiliations:** Department of Chemistry, 14736 Texas A&M University, College Station, Texas 77843, United States

**Keywords:** Exciton-Polaritons, Exciton-Polariton transport, Exciton-Polariton dispersion, Light-Matter Interactions, Cavity Quantum Electrodynamics

## Abstract

We develop an analytical, microscopic theory to describe polaron-polariton dispersion, formed by hybridizing excitons, photons, and phonons, as well as their coherent dynamics inside optical cavities. Starting from a microscopic light-matter Hamiltonian, we derive a simple analytical model by employing a nonperturbative treatment of the phonon and photon couplings to excitons. Within our theoretical framework, phonons are treated as classical fields, which are then quantized via the Floquet formalism. We show that, to a good approximation, the entire polaron-polariton system can be described with a band picture despite the phonons breaking translational symmetry. Our theory also sheds light on the long-lived coherent ballistic motion of exciton-polaritons with high excitonic character that propagate with group velocities lower than expected from pure exciton-polariton bands, offering a microscopic explanation for these puzzling experimental observations.

## Graphical abstract

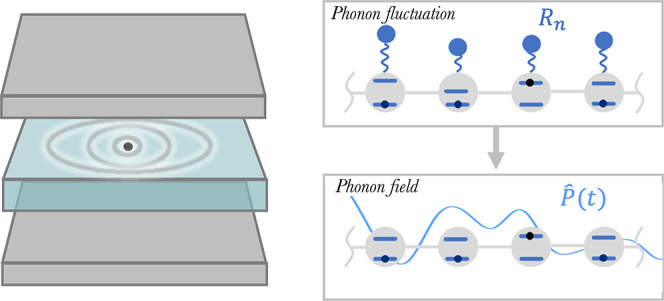

Coupling quantized electromagnetic radiation to excitons forms exciton-polaritons (EPs), a hybrid photon-matter quasi-particle,^1–7^ that demonstrates a wide range of exotic phenomena,^8–16^ including enhanced transport surpassing the inherent limits of bare-exciton transport.^5,17–22^ This extraordinary phenomenon, namely cavity-enhanced exciton transport, demonstrates the unique nature of exciton-polaritons, redefining the traditional paradigms of energy transport with possible applications in quantum information science and chemical reactivity.^5,7,23^

A superposition of neighboring exciton states in reciprocal space leads to coherent ballistic propagation with a group velocity equal to the slope of the band structure in the absence of dissipation.^24^ Phonons, which are intrinsic to materials, break the translational symmetry of an excitonic system, leading to phonon-induced decoherence and incoherent diffusive motion.^25–28^ At the same time, it is expected that the coherent ballistic motion of exciton-polaritons will exhibit group velocities matching the exciton-polariton dispersion for times less than the decoherence lifetime.^7,29,30^ Interestingly, recent experiments^18,19,31,32^ indicate that exciton-polaritons with significantly high excitonic character (up to ∼50% excitonic) show long-lived coherent ballistic motion (up to hundreds of femtoseconds)^17–19,33^ with group velocities lower than the slopes of the exciton-polariton band structure extracted from the linear spectra.^5,18,19,32^ Despite many recent insightful theoretical works on exciton-polariton dynamics,^18,34–40^ a full microscopic understanding of this extraordinary phenomenon has remained elusive. This includes a recent study^37^ that computed group-velocity renormalization within a perturbative framework; however, it does not reproduce the correct polaritonic dispersion nor establish its connection to the experimentally observed group velocities and does not provide an explanation to the long-lived coherence of polaritons.

Here, we introduce a new theoretical framework to understand the complex polariton dispersion formed by hybridizing excitons, photons, and phonons, as well as investigating their coherent dynamics inside optical cavities. Given the intractable nature of the full quantum mechanical problem, we introduce a convenient picture where exciton-polaritons are embedded in a classical phonon field. We quantize this phonon field using the Floquet formalism to derive an analytical model exhibiting *translational* symmetry to a good approximation (near *k* → 0 where the experiments also operate^18,19,41^ ), allowing for coherent motion. This analytical model produces an extremely accurate description of exciton-polariton dispersion when compared to the angle-resolved polariton spectra obtained using a mixed quantum-classical approach.^18,34–37^ Using our model, we show that the presence of phonons introduces vibronic structure in the exciton-polariton dispersion, which we refer to as the polaron-polariton dispersion. We show that this vibronic structure, which is beyond the scope of a perturbative treatment of phonon interactions implemented in recent work,^37^ is responsible for a renormalization of the group velocity and despite a strong interaction with phonons, an effective band structure model can be extracted. Our theory not only serves as a convenient analytical model to understand polariton spectra but also provides new insights into the interplay between phonons and exciton-polaritons.

We consider a generalized multimode Holstein-Tavis-Cummings Hamiltonian,^7,8,34,42^ which describes an exciton-polariton system beyond the long-wavelength approximation, interacting with phonons and is written as

1 $${Ĥ}_{LM}=\underset{n}{\sum }{\mathrm{X̂}}_{n}^{†}{\mathrm{X̂}}_{n}\varepsilon_{0}+\underset{n,k}{\sum }\frac{\Omega_{k}}{\sqrt{N}}\left[{â}_{k}^{†}{\mathrm{X̂}}_{n}e^{-ik\cdot r_{n}}+{â}_{k}{\mathrm{X̂}}_{n}^{†}e^{ik\cdot r_{n}}\right]+\underset{k}{\sum }{â}_{k}^{†}{â}_{k}\omega_{c}\left(k\right)+\tau \underset{n}{\sum }\left({\mathrm{X̂}}_{n}^{†}{\mathrm{X̂}}_{n+1}+{\mathrm{X̂}}_{n+1}^{†}{\mathrm{X̂}}_{n}\right)+\underset{n,j}{\sum }\gamma_{j}{\mathrm{X̂}}_{n}^{†}{\mathrm{X̂}}_{n}{\mathrm{R̂}}_{n,j}+\underset{n,j}{\sum }\frac{{\mathrm{P̂}}_{n,j}^{2}}{2}+\frac{1}{2}\omega_{j}^{2}{\mathrm{R̂}}_{n,j}^{2}={Ĥ}_{EP}+\underset{n,j}{\sum }\gamma_{j}{\mathrm{X̂}}_{n}^{†}{\mathrm{X̂}}_{n}{\mathrm{R̂}}_{n,j}+\underset{n,j}{\sum }\frac{{\mathrm{P̂}}_{n,j}^{2}}{2}+\frac{1}{2}\omega_{j}^{2}{\mathrm{R̂}}_{n,j}^{2}$$

Here ${\mathrm{X̂}}_{n}^{†}$ $\left({â}_{k}^{†}\right)$ creates an excitation (photon) at site *n* (mode *k*), *R*_*n*,*j*_ (*P*_*n*,*j*_) is the position (momentum) operator for the *j*th phonon mode at the *n*th site, and ${Ĥ}_{EP}$ in the last line is the pure exciton-photon Hamiltonian. Here *ε*_0_ is the on-site energy with each site located at *r*_*n*_ = *a* · *n* with *r*_*n*+*N*_ = *r*_*n*_, where *N* is the total number of sites, *a* is the lattice constant, and τ is the hopping parameter. Further, {ω_*j*_} and {γ_*j*_} are the phonon frequencies and its coupling to a excitonic site which are sampled from a spectral density $J\left(\omega \right)=\underset{j}{\sum }\frac{\gamma_{j}^{2}}{\omega_{j}}\delta \left(\omega -\omega_{j}\right)$ . Finally, ω_*c*_(*k*) and $\Omega_{k}=\Omega \sqrt{\omega_{c}\left(0\right)/\omega_{c}\left(k\right)}$ are the photon frequency and exciton-photon couplings, respectively. Additionally, we restrict our system to the single excited subspace, and do not consider nonlinear interactions or many-body effects.^43^ Further details are provided in the Supporting Information (SI). Notably, the phonon degrees of freedom break the translational symmetry of the exciton-polariton system. Nevertheless, we demonstrate that a quasi-band structure framework can be employed, effectively capturing the complex ballistic transport of exciton-polaritons.

Direct (analytical or numerical) quantum mechanical treatment of this light-matter Hamiltonian is a formidable task given that polaritonic dispersion can only be obtained when using *N* ∼ 10^5^ sites for experimentally relevant values of the lattice constant *a* (chosen here to be 1.2 nm). To solve this intractable problem, we employ a mixed-quantum-classical approach, namely the mean-field Ehrenfest (MFE) method,^7,44,45^ that is known to accurately reproduce quantum vibronic structure in optical spectra in a single-site exciton–phonon model,^46,47^ despite the classical treatment of phonons. Note that the primary drawback of the MFE approach is its violation of detailed balance,^48^ which leads to inaccurate long-time population dynamics, even though it yields accurate correlation functions at short times.^46,49,50^ In the present work, we compute only those correlation functions that require short-time propagation and focus exclusively on dynamics up to roughly 200 fs. In the SI, we compare numerically exact results for few-site models (up to seven sites) with the MFE approach, demonstrating that this simple mixed quantum–classical method produces results of reasonable accuracy, in agreement with previous studies.^46,49–51^ In spite of the quasi-classical treatment of the phonons, MFE can capture the quantum vibronic progression which becomes exact for low frequencies or at high temperatures.^52^

These calculations reveal that while the relative peak positions of the photonic spectral function are reproduced by the MFE with reasonable accuracy, the corresponding peak heights (intensities) can deviate, albeit without any qualitative discrepancies. Within this approach, the phonon modes are treated quasi-classically, i.e. $\left\{{\mathrm{R̂}}_{n,j},{\mathrm{P̂}}_{n,j}\right\}\to \left\{R_{n,j},P_{n,j}\right\}$ , while the photonic and excitonic parts are propagated quantum mechanically using the *polaritonic* Hamiltonian ${Ĥ}_{pl}\left(R\right)={Ĥ}_{LM}-\frac{1}{2}\underset{n,j}{\sum }\left(P_{n,j}^{2}-\omega_{j}^{2}R_{n,j}^{2}\right)$ . The equations of motion in the MFE approach (in atomic units) are written as

2 $$i\left|\mathrm{\Psi ̇}\left(t\right)\right\rangle ={Ĥ}_{pl}\left(R\right)\left|\Psi \left(t\right)\right\rangle$$

3 $${\mathrm{R̈}}_{n,j}\left(t\right)={Ṗ}_{n,j}\left(t\right)=-\left\langle \Psi \left(t\right)\left|\frac{{Ĥ}_{LM}\left(R\right)}{R_{n,j}}\right|\Psi \left(t\right)\right\rangle$$

The initial nuclei positions and momentums {*R*_*n*,*j*_(0), *P*_*n*,*j*_(0)} are sampled from a Wigner distribution (see details in the SI), and an expectation value of an operator Â is computed as $\left\langle Â\right\rangle \approx {\left\langle \left\langle \Psi \left(t\right)\right|Â\left|\Psi \left(t\right)\right\rangle \right\rangle }_{MFE}$ , where ⟨···⟩_MFE_ indicates averaging over realizations of initial nuclear coordinates {*R*_*n*,*j*_(0), *P*_*n*,*j*_(0)}.

The angle-resolved photonic spectral function *I*(ω, *k*) is obtained by computing

I(ω,k)=Re[limT→∞⁡∫0Tdt⁡eiωt⟨⟨1k|Ψ(t)⟩⟩MFE·cos(πt/2T)]

where $\left|\Psi \left(0\right)\right\rangle ={â}_{k}^{†}\left|\mathrm{0̅}\right\rangle =\left|1_{k}\right\rangle$ , with |0̅⟩ as the vacuum state. Note that we have included the term cos(πt/2T) to suppress spurious Gibbs oscillations. Our numerical results, presented in Figure 1, illustrates the emergence of complex vibronic structure in the momentum-resolved polaritonic spectra in the presence of phonon modes. As can be seen in these figures, despite the absence of a strict translational symmetry, the angle-resolved spectra suggests the existence of a quasi-band of polaron-polaritons. Such vibronic structure in exciton-polariton bands has been seen in recent experiments.^16,53–55^ Below, we derive the analytical forms of these quasi-bands with details provided in the Supporting Information.

**Figure 1:**
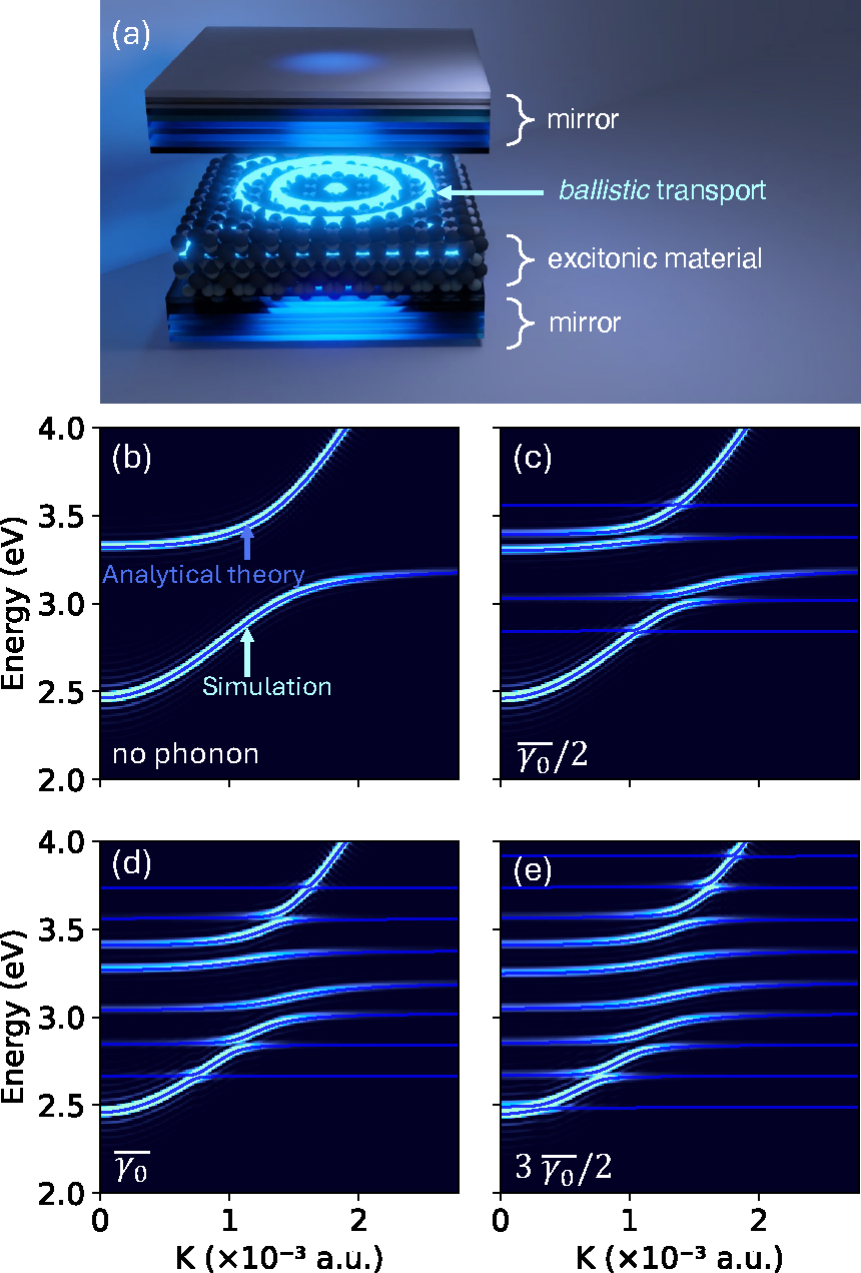
(a) Schematic illustration model of exciton-polariton transport inside an optical cavity. (b)–(e) Polaron-polariton dispersion obtained with MFE (simulation) compared to our analytical theory (at room temperature *T* = 300 K) with different phonon couplings: (b) γ_0_ = 0, (c) $\gamma_{0}=\overset{®}{\gamma_{0}\;}/2$ , (d) $\gamma_{0}=\overset{®}{\gamma_{0}\;}$ , and (e) $\gamma_{0}=3\overset{®}{\gamma_{0}\;}/2$ , where $\overset{®}{\gamma_{0}\;}=5.85\times 10^{-4}$ a.u. Further, we use Ω = 2400 cm^–1^, *N* = 40001, τ = 0, ω_*c*_(0) = 2.58 eV, and *ε*_0_ = 3.2 eV.

To obtain an analytical expression for these polaron-polariton (quasi) bands, we first make the classical path approximation,^27,56,57^ such that ${\mathrm{R̈}}_{n,j}\left(t\right)\approx -\omega_{j}^{2}R_{n,j}\left(t\right)$ with

4 $$R_{n,j}\left(t\right)\approx R_{n,j}\left(0\right)\cos\omega_{j}t+\frac{1}{\omega_{j}}P_{n,j}\left(0\right)\sin\omega_{j}t$$

With this analytical expression of *R*_*n*,*j*_(*t*), the dynamics of the exciton-polariton wave function |Ψ⟩ can be thought to be evolving under the time-periodic Hamiltonian ${Ĥ}_{pl}\left(t\right)$ expressed as

5 $${Ĥ}_{pl}\left(t\right)={Ĥ}_{EP}+\underset{j}{\sum }\left({\mathrm{P̂}}_{j}e^{i\omega_{j}t}+{\mathrm{P̂}}_{j}^{†}e^{-i\omega_{j}t}\right)$$

where ${\mathrm{P̂}}_{j}=\underset{n}{\sum }\gamma_{j}{\mathrm{X̂}}_{n}^{†}{\mathrm{X̂}}_{n}Z_{n,j}$ describes the interaction to a classical phonon field with $Z_{n,j}=\frac{R_{n,j}\left(0\right)}{2}+\frac{P_{n,j}\left(0\right)}{2i\omega_{j}}$ . Note that the classical path approximation remains valid for the exciton-polariton dynamics confined to the single excited subspace (relevant to the present study) and that it can break down at high excitations where many-body effect can also persist.^43^

Notice the similarity between ${Ĥ}_{pl}\left(t\right)$ and the typical laser-matter Hamiltonian, with phonon degrees of freedom (or molecular vibrations) in our system playing the same role as a laser field. We adapt the Floquet formalism^58–63^ and rewrite ${Ĥ}_{pl}\left(t\right)$ in an extended space (so-called Sambe space) as a time-independent Hamiltonian ${Ĥ}_{F}$ such that

6 Ĥpl(t)→ĤF=limM→∞⁡∑α,β|β⟩⟨β|ĤF|α⟩⟨α|⁣with⁡⁡{|α⟩,|β⟩}∈{X̂n†∏j(Bj^†)M+mj(M+mj)!|0̅⟩,âk†∏j(Bj^†)M+mj(M+mj)!|0̅⟩}

Here, ${Ĥ}_{F}$ denotes the quantized Floquet Hamiltonian expressed in the extended basis {|α⟩, |β⟩}, which includes photonic or excitonic states carrying *m*_*j*_ excitations in the *collective phonon field* (in the limit *M* → *∞*). We have introduced the bosonic operator ${\mathrm{B̂}}_{j}^{†}$ to create an excitation in this collective phonon field of frequency ω_*j*_. Note that the typical multimode Floquet formalism becomes intractable for noncommensurate frequencies, since it formally requires an infinite basis in the extended Hilbert space. However, owing to the structure of light–matter coupling used here, we find that only a finite and computationally tractactble number of Floquet states is needed to achieve convergence.

The time-independent Hamiltonian in the Sambe-space, ${Ĥ}_{F}$ is expressed as

$${Ĥ}_{F}=\underset{n,j}{\sum }\left(\varepsilon_{0}+\frac{\gamma_{j}}{\sqrt{M}}\left(Z_{n,j}{\mathrm{B̂}}_{j}+Z_{n,j}^{*}{\mathrm{B̂}}_{j}^{†}\right)\right){\mathrm{X̂}}_{n}^{†}{\mathrm{X̂}}_{n}+\tau \underset{n}{\sum }\left({\mathrm{X̂}}_{n}^{†}{\mathrm{X̂}}_{n+1}+{\mathrm{X̂}}_{n+1}^{†}{\mathrm{X̂}}_{n}\right)+\underset{j}{\sum }\omega_{j}\left({\mathrm{B̂}}_{j}^{†}{\mathrm{B̂}}_{j}-M\right)+\underset{k}{\sum }{â}_{k}^{†}{â}_{k}\omega_{c}\left(k\right)+\underset{n,k}{\sum }\frac{\Omega_{k}}{\sqrt{N}}\left({â}_{k}^{†}{\mathrm{X̂}}_{n}e^{-ik\cdot r_{n}}+{â}_{k}{\mathrm{X̂}}_{n}^{†}e^{ik\cdot r_{n}}\right)$$ 7

Next, we perform a polaron transformation on ${Ĥ}_{F}$ using the operator ${Û}_{D}$ defined as

8 $${Û}_{D}=\underset{n,j}{\prod }\exp\left[\left(Z_{n,j}^{*}{\mathrm{B̂}}_{j}^{†}-Z_{n,j}{\mathrm{B̂}}_{j}\right)\frac{\gamma_{j}{\mathrm{X̂}}_{n}^{†}{\mathrm{X̂}}_{n}}{\omega_{j}\sqrt{M}}\right]$$

to obtain ${Ĥ}_{F}^{\prime }={Û}_{D}^{†}{Ĥ}_{F}{Û}_{D}$ that is explicitly written as

9 $${Ĥ}_{F}^{\prime }=\underset{n}{\sum }\varepsilon_{0}{\mathrm{X̂}}_{n}^{†}{\mathrm{X̂}}_{n}+\underset{j}{\sum }\left({\mathrm{B̂}}_{j}^{†}{\mathrm{B̂}}_{j}-M\right)\omega_{j}+\underset{k}{\sum }{â}_{k}^{†}{â}_{k}\omega_{c}\left(k\right)+\tau \underset{n}{\sum }\left({\mathrm{X̂}}_{n}^{†}{\mathrm{X̂}}_{n+1}\underset{j}{\prod }\exp\left[\gamma_{j}\frac{\Delta Z_{n,j}{\mathrm{B̂}}_{j}-\Delta Z_{n,j}^{*}{\mathrm{B̂}}_{j}^{†}}{\omega_{j}\sqrt{M}}\right]+h.c.\right)+\underset{n,k}{\sum }\frac{\Omega_{k}}{\sqrt{N}}\left({â}_{k}^{†}{\mathrm{X̂}}_{n}\underset{j}{\prod }\exp\left[\frac{\gamma_{j}\left(Z_{n,j}{\mathrm{B̂}}_{j}-Z_{n,j}^{*}{\mathrm{B̂}}_{j}^{†}\right)}{\omega_{j}\sqrt{M}}-ik\cdot r_{n}\right]+h.c.\right)$$

Here Δ*Z*_*n*,*j*_ = *Z*_*n*+1,*j*_ – *Z*_*n*,*j*_. Below, we make further simplifications to obtain a convenient expression to obtain the polaron polariton quasi-bands, with details provided in the SI. First, we restrict the extended subspace to include only *m*_*j*_ = 0 when considering photonic excitations such that

{|α⟩,|β⟩}∈{X̂n†∏j(Bj^†)M+mj(M+mj)!|0̅⟩,âk†∏j(Bj^†)MM!|0̅⟩}

Second, we introduce the following *effective* reciprocal (phonon-dressed) exciton operators

10 $${Ŷ}_{k,\mathrm{m⃗}}^{†}=\underset{M\to \infty }{\lim}\underset{n}{\sum }e^{-ik\cdot r_{n}}{\mathrm{X̂}}_{n}^{†}\underset{j}{\prod }\frac{Q_{0m_{j}}^{\left[j\right]}\left(Z_{n,j}\right)}{\sqrt{S_{m_{j}}^{\left[j\right]}\left(M+m_{j}\right)!}}{\left({\mathrm{B̂}}_{j}^{†}\right)}^{M+m_{j}}$$

where $\mathrm{m⃗}=\left(m_{0},m_{1},...\right)$ , $S_{m_{j}}^{\left[j\right]}=\underset{n}{\sum }|Q_{0m_{j}}^{\left[j\right]}\left(Z_{n,j}\right){|}^{2}$ is a normalization factor and $Q_{0m_{j}}^{\left[j\right]}\left(Z_{n,j}\right)$ is the overlap between infinitely excited displaced Fock states of the *j*th phonon mode written as

11 Q0mj[j](Z̃)=limM→∞⟨M|exp[−γjωjM(Z̃Bj^−Z̃*B̂j†)]|M+mj⟩

where Z̃ is a complex number. In the SI, we show that $\left\langle \mathrm{0̅}\right|{Ŷ}_{k,{\mathrm{m⃗}}^{\prime }}{Ŷ}_{k,\mathrm{m⃗}}^{†}\left|\mathrm{0̅}\right\rangle \approx \delta_{\mathrm{m⃗},{\mathrm{m⃗}}^{\prime }}$ (for *k* → 0), using which we write

12 $${Ĥ}_{F}^{\prime }\approx \underset{k}{\sum }[{â}_{k}^{†}{â}_{k}\omega_{c}\left(k\right)+\underset{\mathrm{m⃗},j}{\sum }\left(\mathrm{\varepsilon ̅}+m_{j}\omega_{j}\right){Ŷ}_{k,\mathrm{m⃗}}^{†}{Ŷ}_{k,\mathrm{m⃗}}+\underset{\mathrm{m⃗}}{\sum }\underset{j}{\prod }\sqrt{\frac{S_{m_{j}}^{\left[j\right]}}{N}}\Omega_{k}\left({Ŷ}_{k,\mathrm{m⃗}}^{†}{â}_{k}+{â}_{k}^{†}{Ŷ}_{k,\mathrm{m⃗}}\right)]=\underset{k}{\sum }{Ĥ}_{k}$$

where $\mathrm{\varepsilon ̅}=\varepsilon_{0}+2\tau \cdot \xi_{0}$ with $\xi_{0}=\underset{n}{\sum }\underset{j}{\prod }Q_{00}^{\left[j\right]}\left(\Delta Z_{n,j}\right)\times Q_{0m_{j}}^{\left[j\right]}\left(Z_{n,j}\right)\times Q_{0m_{j}}^{\left[j\right]}\left(Z_{n+1,j}\right)/S_{m_{j}}^{\left[j\right]}$ describing a phonon induced suppression of the hopping term τ. Here ${Ĥ}_{F}^{\prime }$ is block diagonal in *k*, thus allowing us to extract the phonon-modified exciton-polariton (or equivalently polaron-polariton) dispersion. In the case of a single phonon-mode per site with $J\left(\omega \right)=\frac{\gamma_{0}^{2}}{\omega_{0}}\delta \left(\omega -\omega_{0}\right)$ , polaron-polariton (quasi) bands are obtained by diagonalizing a simplified ${Ĥ}_{k}$ matrix written as

13 $${Ĥ}_{k}=\left[\begin{array}{lllllll} \ddots & \vdots & \vdots & \vdots & & \vdots \\ ... & \mathrm{\varepsilon ̅}-\omega_{0} & 0 & 0 & ... & \sqrt{\frac{S_{-1}^{\left[0\right]}}{N}}\Omega_{k}\\ ... & 0 & \mathrm{\varepsilon ̅} & 0 & ... & \sqrt{\frac{S_{0}^{\left[0\right]}}{N}}\Omega_{k}\\ ... & 0 & 0 & \mathrm{\varepsilon ̅}+\omega_{0} & ... & \sqrt{\frac{S_{1}^{\left[0\right]}}{N}}\Omega_{k}\\ & \vdots & \vdots & \vdots & \ddots & \vdots \\ ... & \sqrt{\frac{S_{-1}^{\left[0\right]}}{N}}\Omega_{k} & \sqrt{\frac{S_{0}^{\left[0\right]}}{N}}\Omega_{k} & \sqrt{\frac{S_{1}^{\left[0\right]}}{N}}\Omega_{k} & ... & \omega_{c}\left(k\right) & \end{array}\right]$$

Overall, we find that phonon interactions modify the exciton polariton bands in specifically two ways.

First, it introduces vibronic states, which are effectively captured via the collective phonon field excitations via ${\mathrm{B̂}}_{j}^{†}$ within our mixed quantum-classical framework, coupling to the photonic bands forming a Rabi-Splitting of $\Omega_{k}=\underset{j}{\prod }\sqrt{S_{m_{j}}^{\left[j\right]}/N}$ . This can be calculated by integrating the squared overlap $\underset{j}{\prod }{\left[Q_{m0}^{\left[j\right]}\left(Z_{n,j}\right)\right]}^{2}$ over the Wigner distribution of {*R*_*n*,*j*_(0), *P*_*n*,*j*_(0)} such that

14 $$S_{m_{j}}^{\left[j\right]}=\underset{M\to \infty }{\lim}2\tanh\left(\frac{\beta \omega_{j}}{2}\right)\int_{-\infty }^{\infty }dz\frac{M!}{\left(M+m_{j}\right)!}{\left(\frac{\gamma_{j}^{2}z^{2}}{\omega_{j}^{2}M}\right)}^{m_{j}}\times \exp\left[-\left(4\omega_{j}\tanh\left(\frac{\beta \omega_{j}}{2}\right)+\frac{z_{j}^{2}\gamma_{j}^{4}}{4\omega_{j}^{4}M^{2}}\right)z^{2}\right]{\left[L_{M}^{\left(m_{j}\right)}\left(\frac{\gamma_{j}^{2}z^{2}}{\omega_{j}^{2}M}\right)\right]}^{2}$$

Here, $\beta ={\left(k_{B}T\right)}^{-1}$ with *k*_B_ the Boltzmann constant and *T* = 300 K is the temperature. Further, $L_{M}^{\left(m_{j}\right)}$ is the associated Laguerre polynomial. Note that the model presented in eq 12–14, is structurally different from previously proposed models for polaritonic spectra^64–66^ that incorporated the vibronic progression in the polariton dispersion in an ad-hoc manner.

Second, the phonons renormalize the hopping term τ, shifting up the excitonic energy near *k* → 0 as expected. In the following, we will focus on the vibronic structure and its implication for the polariton dispersion and set τ = 0 (relevant for molecular exciton-polaritons) and consider a single mode per site with $J\left(\omega \right)=\frac{\gamma_{0}^{2}}{\omega_{0}}\delta \left(\omega -\omega_{0}\right)$ which is adequate for describing various organic molecules that have a rigid structure (such as polycyclic aromatic hydrocarbons). In the SI, we present results when τ ≠ 0 relevant for exciton-polaritons in extended materials. In the SI, we also present results when considering multiple phonon modes per site, described with the spectral density $J\left(\omega \right)=\underset{j}{\sum }\frac{\gamma_{j}^{2}}{\omega_{j}}\delta \left(\omega -\omega_{j}\right)$ , and find similar polaron-polariton dynamics (such as group velocity renormalization) and spectra with the same level of accuracy of our analytical approach (as in the main-text). This illustrates the applicability of our approach in a wider range of model systems.

Figure 1 presents the angle-resolved polariton spectra comparing with polariton (quasi) bands obtained using our analytical model presented in eq 13. Figure 1a schematically illustrates an excitonic material placed inside a Fabry-Pérot cavity exhibiting ballistic transport of polaritons. The angle-resolved polariton spectra in the absence of phonons, presented in Figure 1b reduce to the two-band coupled oscillator model used widely.^3,7,8^ Our theoretical model (solid blue lines) reduces to this two-band model when setting γ_0_ = 0 (no phonon interactions), thus exactly reproducing the polaritonic spectra in Figure 1b.

Figure 1c-e presents the polariton spectra obtained from our mixed-quantum classical simulation at various phonon couplings γ_0_, comparing them to the predictions of our analytical model. The vibronic structure shown in Figure 1c-e has been reported in multiple experimental works^16,41,53–55^ with our analytical model offering a microscopic understanding of the polariton spectra. Our nonperturbative treatment of phonon couplings is essential for capturing these vibronic features. We emphasize that, when considering multiple phonon modes per site, we find that the vibronic structure diminishes (see Figures S7 and S8), as is the case for molecular spectra. Thus, whether or not these vibronic structures will show up in the polariton spectra depends entirely on the spectral density of the system under consideration. Our theory operates conveniently in all of such circumstances.

At relatively small phonon coupling $\gamma_{0}={\mathrm{\gamma ̅}}_{0}/2$ (we set ${\mathrm{\gamma ̅}}_{0}=5.85\times 10^{-4}$ a.u.), we observe a clear phonon-induced splitting of the lower polariton band around 3 eV. Note that with increasing phonon coupling, the exciton on-site energy minima along the phonon displacement coordinate is shifted down by the reorganization energy $\lambda =\frac{1}{2}\frac{\gamma_{0}^{2}}{\omega_{0}^{2}}$ . As a result, increasing γ_0_ also leads to the formation of phonon-induced splitting at progressively lower energy. At the same time, increasing γ_0_ also introduces more splitting. This is because the increase in the displacement of the phonon field leads to more sizable overlap between the photonic states and excitonic states with *m* phonon field excitation or de-excitation in the Floquet picture employed here.

With the success in obtaining the phonon-modified polariton bands (which we refer to as polaron polariton bands) using our analytical model, in Figure 2 we use it to obtain the group velocities and provide insights into the coherent propagation of polaron-polaritons. In the absence of phonons, a coherent superposition of neighboring wave vectors in the momentum space, e.g. $\left|\Psi \right\rangle =\underset{F\delta k\to 0}{\lim}\frac{1}{\sqrt{F}}\overset{F}{\underset{n=1}{\sum }}\left|k+n\delta k\right\rangle$ in an extended system, leads to coherent ballistic propagation with a group velocity equal to the slope of the band structure *dE*/*dk*, known as the group velocity. On the other hand, in the presence of phonons, phonon-induced decoherence leads to incoherent diffusive motion. Therefore, it is expected that exciton-polaritons, depending on the extent of their material character, will lead to short-time (for times less than the decoherence lifetime) coherent ballistic motion with group velocities matching the exciton-polariton dispersion. Experimental results, however, indicate that exciton-polaritons with significantly high excitonic character (up to ∼ 50% excitonic) show long-lived coherent ballistic motion (up to hundreds of femtoseconds) with group velocities lower than the slopes of the exciton-polariton band structure. Our theory provides a plausible explanation for this 2-fold mystery and provides new microscopic insights into this extraordinary phenomenon.

**Figure 2:**
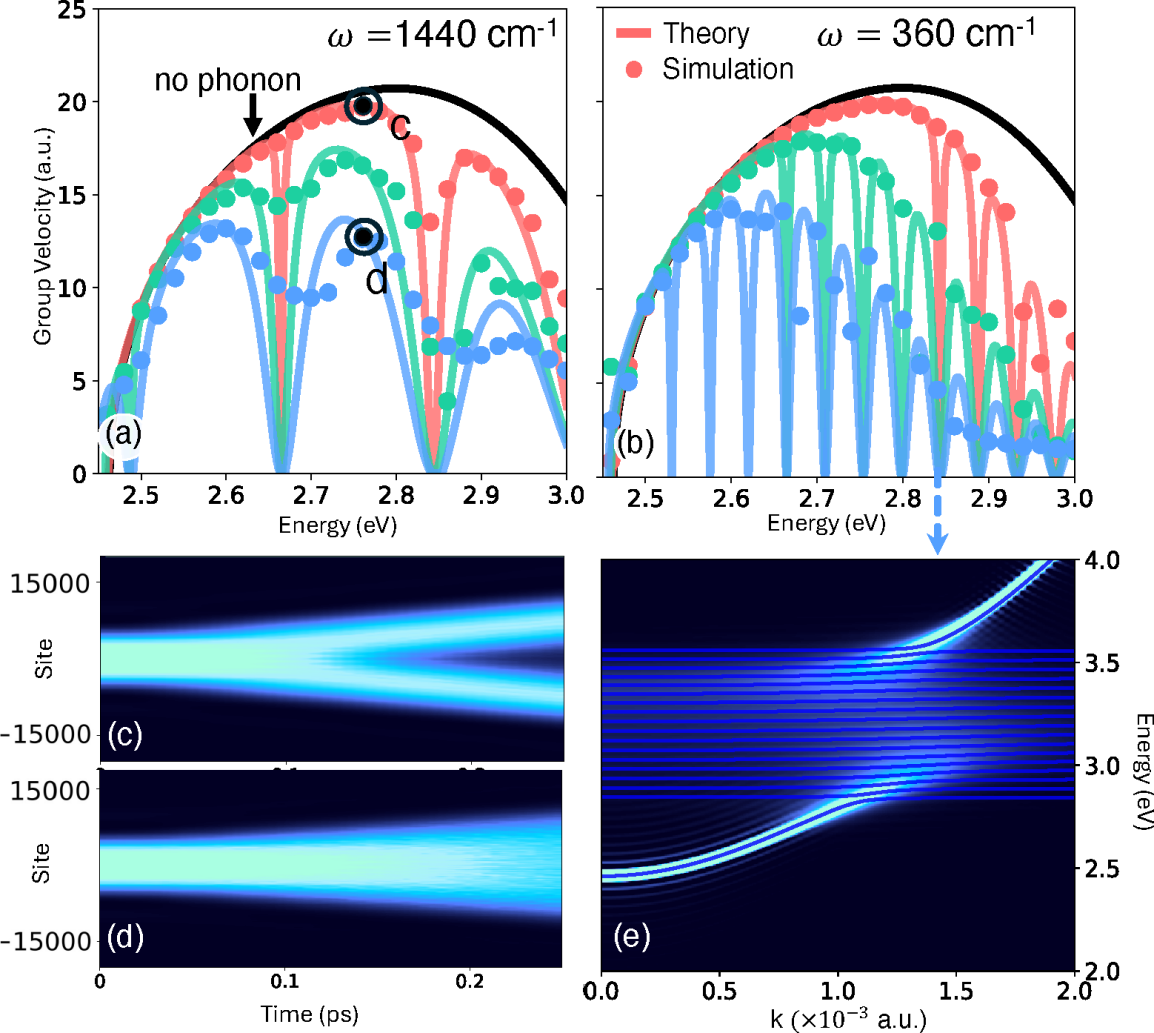
(a)-(b) Phonon modified exciton polariton group velocities obtained using direct mixed quantum classical simulations (filled circles) compared to our analytical theory (solid lines) at a phonon frequency (a) ω_0_ = 1440 cm^–1^ and (b) ω_0_ = 360 cm^–1^ with various phonon couplings. In panel (a) we consider γ_0_ = 0 (black), $\gamma_{0}={\mathrm{\gamma ̅}}_{0}/2$ (red), $\gamma_{0}={\mathrm{\gamma ̅}}_{0}$ (green), and $\gamma_{0}=3{\mathrm{\gamma ̅}}_{0}/2$ (blue) with $\overset{®}{\gamma_{0}\;}=5.85\times 10^{-4}$ a.u. In panel (b) we consider γ_0_ = 0 (black), $\gamma_{0}={\mathrm{\gamma ̅}}_{0}/2$ (red), $\gamma_{0}={\mathrm{\gamma ̅}}_{0}$ (green) and $\gamma_{0}=3{\mathrm{\gamma ̅}}_{0}/2$ (blue) with ${\mathrm{\gamma ̅}}_{0}=1.46\times 10^{-4}$ a.u. (c)-(d) Time-dependent polariton density in the (c) absence and (d) presence of phonon couplings. (e) Polaron-polariton dispersion computed using MFE and our analytical approach (blue solid lines) for the same parameters as in the blue curve in (b). Further, we use Ω = 2400 cm^–1^, τ = 0, ω_*c*_(0) = 2.58 eV, and *ε*_0_ = 3.2 eV and *N* = 40001 in panel (a) and *N* = 30001 for (b).

Figure 2a-b presents the polariton group velocity obtained from our analytical model (solid lines), comparing it to the group velocities obtained by performing direct mixed quantum-classical simulations (filled circles) at two different phonon frequencies and various phonon couplings. Figure 2c-d presents time-dependent polaritonic density

$$\rho_{n}\left(t\right)=\left\langle \left\langle \Psi \left(t\right)\right|{\mathrm{X̂}}_{n}^{†}{\mathrm{X̂}}_{n}+{â}_{n}^{†}{â}_{n}\right|\Psi {\left(t\right)\rangle \rangle }_{MFE}$$

in the presence of (d) and absence of (c) phonon couplings. We have prepared the initial exciton-polariton wave function as a linear combination of polariton states within an energy window Δ*E* centered at an excitation energy *E*_0_, such that |Ψ(0)⟩ = *∑c*_*j*_|*E*_*j*_⟩ with *E*_0_ – Δ*E*/2 < *E*_*j*_ < *E*_0_ + Δ*E*/2 and |*E*_*j*_⟩ as the eigenstates of ${Ĥ}_{\mathrm{EP}}$ . In both cases, we observe a ballistic propagation suggested by the linear expansion of the wavefront in time, with (d) propagating relatively slowly compared to (c). We extract the group velocities from these wavefronts, which are presented in Figure 2a-b (filled circles) and are compared to the predictions of our analytical model.

At higher phonon frequencies, the vibronic structure in the dispersion directly results in an oscillatory behavior in the group velocity with troughs separated by the phonon frequency ω_0_. At lower phonon frequencies, such as in Figure 2b, the oscillatory structure is almost absent as the peaks in the analytical theory pack closer. Figure 2e presents the angle-resolved spectra at ω_0_ = 360 cm^–1^ where the vibronic peaks are no longer visible due to the finite line width of the optical spectra leading to a broadening at *k*/*a* ≈ 1.5 × 10^–3^ a.u. (see Figure S9). Therefore, even though the vibronic structure is not visible in polaritonic spectra, it results in a renormalization of the group velocity. This phenomenon has been observed experimentally,^18,19^ with our theory providing a clear theoretical explanation.

In both scenarios, however, the observed group velocities are always lower, due to the formation of the polaron-polariton (quasi) bands that have flatter slopes, due to the contribution of the flat effective exciton bands ${Ŷ}_{k,m}$ , compared to the bare exciton-polariton dispersion. This renormalization of the exciton-polariton group velocity is induced by the presence of phonons in materials, and even at low phonon frequencies, where the vibronic structure in the angle-resolved spectra may be hidden due to various sources of dissipation (such as cavity loss), the quasi-bands lead to the renormalization of the group velocity. Overall, our theoretical model correctly captures the complex ballistic propagation of exciton-polaritons in the presence of phonon interactions and introduces a quasi-band picture that can be adopted to describe and understand the coherent propagation of polaron-polaritons.

Importantly, our work also suggests a microscopic explanation for the relatively long-lived coherent propagation of exciton-polaritons with high exciton character^5,18,19,32^ at room temperature. We hypothesize that the origin of this extraordinary effect is the block diagonal nature of eq 10 where photon modes ${â}_{k}^{†}$ couple to a particular set of *effective* reciprocal (phonon-dressed) excitons ${Ŷ}_{k,m}$ with matching *k*, defined in eq 10. To clearly understand the ramifications of this, consider first a bare excitonic system coupled with phonons under laser driving E(t) that target a subspace K in reciprocal space, which can be written as

15 $${Ĥ}_{X}+{Ĥ}_{laser}=\underset{k}{\sum }{\mathrm{X̂}}_{k}^{†}{\mathrm{X̂}}_{k}\epsilon_{k}+\frac{\gamma_{0}}{\sqrt{2\omega_{0}}}\underset{k,q}{\sum }{\mathrm{X̂}}_{k+q}^{†}{\mathrm{X̂}}_{k}\left({\mathrm{b̂}}_{q}+{\mathrm{b̂}}_{-q}^{†}\right)+\underset{k}{\sum }{\mathrm{b̂}}_{k}^{†}{\mathrm{b̂}}_{k}\omega_{0}+E\left(t\right)\underset{k\in K}{\sum }\left({\mathrm{X̂}}_{k}^{†}+{\mathrm{X̂}}_{k}\right)$$

where ${\mathrm{b̂}}_{q}^{†}=\frac{1}{\sqrt{N}}\underset{n}{\sum }{\mathrm{b̂}}_{n}^{†}e^{iqr_{n}}$ with $\frac{\left({\mathrm{b̂}}_{n}^{†}+{\mathrm{b̂}}_{n}\right)}{\sqrt{2\omega_{0}}}={\mathrm{R̂}}_{n,0}$ , and ϵ_*k*_ = ϵ_0_ + 2τ cos­(*k* · *a*) for the choice of nearest neighbor interactions made here. Despite a laser exclusively targeting the subspace K , the population leaks out to the subspace M=1−K via the phonon-induced scattering term $\frac{\gamma_{0}}{\sqrt{2\omega_{0}}}\underset{k,q}{\sum }{\mathrm{X̂}}_{k+q}^{†}{\mathrm{X̂}}_{k}\left({\mathrm{b̂}}_{q}+{\mathrm{b̂}}_{-q}^{†}\right)$ . In contrast, inside an optical cavity, following our analytical model in eq 12, a driven light-matter hybrid system can be modeled as^67^

16 $${Ĥ}_{LM}+{Ĥ}_{laser}={Ĥ}_{F}^{\prime }+E\left(t\right)\underset{k\in K}{\sum }\left({â}_{k}^{†}+{â}_{k}\right)\approx \underset{k\in K}{\sum }\left[{Ĥ}_{k}+E\left(t\right)\left({â}_{k}^{†}+{â}_{k}\right)\right]+\underset{k\in M}{\sum }{Ĥ}_{k}$$

such that the subspace M and K now remain decoupled. Therefore, light-matter interaction also plays a crucial role in suppressing phonon-induced scattering in the reciprocal space (suppressing Fröhlich scattering of the energetically localized excitation), allowing for relatively long-lived ballistic motion in the time scale of hundreds of femtoseconds. That said, while a quantitative description of the decoherence lifetime associated with the ballistic-to-diffusive transition^18,19,36,68,69^ in exciton-polariton transport is beyond the scope of the present work, extending our framework to capture this behavior is part of our future goals.

In summary, we developed a convenient theoretical framework to understand and predict the angle-resolved polariton spectra in the presence of phonon interactions. Starting from a microscopic Hamiltonian describing the interactions between phonons, excitons, and photons inside an optical cavity, we develop an analytical model that accurately predicts the complex angle-resolved polariton spectra and the group velocities of coherently propagating exciton-polaritons. We derive this analytical model by describing the phonons as time-periodic fields that are nonperturbatively interacting with exciton-polaritons and quantize them using the Floquet formalism that is typically used to describe laser-matter interactions. This work provides a new perspective into the observed renormalization of polaritonic group velocity and their long-lived coherent nature in recent experiments. Finally, we note that the accuracy of the present approach is limited, especially at lower temperatures or when nuclear quantum effect become dominant, due to the mixed-quantum classical treatment of phonons. Thus, an exact open quantum dynamical simulation of exciton–polaritons at room temperature would be highly desirable.

## Supplementary Material



## Notes

Details of the microscopic theory of polaron-polariton dispersion, initialization of the exciton-polariton wave function via Monte Carlo sampling, additional results for comparing to exact diagonalization and quantum perturbation theory, absorption spectra of multiphonon systems, anharmonic and temperature considerations, and finite cavity lifetime, and parameters used for simulations (PDF)
