# Supplementary material for: Microscopic Theory of Polaron-Polariton Dispersion and Propagation
Source: Nano Lett. 2025 Oct 20;25(44):15874–82. doi: 10.1021/acs.nanolett.5c04134 (PMC12593387; doi:10.1021/acs.nanolett.5c04134)
Supplement: Supplementary file 1 [file nl5c04134_si_001.pdf]

Supplementary Information for Microscopic Theory of Polaron-Polariton Dispersion and Propagation

Logan Blackham[†,1], Arshath Manjalingal[†,1], Saeed Rahmanian Koshkaki[1], Arkajit Mandal[1*]
[1]Department of Chemistry, Texas A&M University, College Station, Texas 77843, USA

October 14, 2025

Contents

S1 Details of the Microscopic Theory of Polaron-Polariton Dispersion 2
S1.1 Holstein-Tavis Cummings Hamiltonian and Quantization of Phonon Field . . . 2
S1.2 Polaron Transformation of the Phonon Field . . . 3
S1.3 Analytical Expression of Overlaps . . . 3
S1.4 Polaron-Polariton Quasi-Band Picture in Reciprocal Space . . . 4
S1.5 Orthogonality of the Effective Reciprocal Excitonic states . . . 5
S1.6 Generalization of Phonon-Polariton Theory to Incorporate Angular Molecular Disorder . . . 5

S2 Initialization of the Exciton-Polariton Wavefunction via Monte Carlo Sampling 6

S3 Additional Results 6
S3.1 Comparing to Exact Diagonalization . . . 6
S3.2 Comparing to Quantum Perturbative Theory . . . 7
S3.3 Absorption Spectra of Multimode Exciton-Polariton Systems . . . 7
S3.4 Anharmonic Considerations . . . 9
S3.5 Temperature Considerations . . . 9
S3.6 Finite Cavity Lifetime . . . 10

S4 Parameters Used For Simulation 10

*Corresponding Author: mandal@tamu, † Equal contribution

S1 Details of the Microscopic Theory of Polaron-Polariton Dispersion

S1.1 Holstein-Tavis Cummings Hamiltonian and Quantization of Phonon Field

We start from the generalized multi-mode Holstein-Tavis-Cummings Hamiltonian, which can be rigorously derived from a non-relativistic QED Hamiltonian [1, 2, 3], which in the atomic units is written as

$$\begin{aligned}\hat{H}_{\rm LM} = &\sum_{n,j}\gamma_j \hat{X}_n^\dagger \hat{X}_n \hat{R}_{n,j} + \sum_n \hat{X}_n^\dagger \hat{X}_n \varepsilon_0 + \tau \sum_n (\hat{X}_n^\dagger \hat{X}_{n+1} + \hat{X}_{n+1}^\dagger \hat{X}_n) + \\ &\sum_k \hat{a}_k^\dagger \hat{a}_k \omega_c(k) + \sum_{n,k}\frac{\Omega_k}{\sqrt{N}}\left[\hat{a}_k^\dagger \hat{X}_n e^{-ik_x\cdot r_n} + \hat{a}_k \hat{X}_n^\dagger e^{ik_x\cdot r_n}\right]\sin(k_y\cdot r_n) \\ &+ \sum_{n,j}\frac{\hat{P}_{n,j}^2}{2} + \sum_{n,j}\frac{1}{2}\omega_j^2 \hat{R}_{n,j}^2,\end{aligned} \tag{S1}$$

where γ_j is the relative exciton-phonon coupling strength for j-th phonon mode, ε_0 is the on site energy of the excitonic state, τ is the hopping term, N as the number of sites, and $\omega_c(k)$ is the photon frequency of mode k. Here, $\Omega_k/\sqrt{N}$ is the light-matter coupling assuming only the TE polarization couples to the exciton where $\Omega_k = \Omega\sqrt{\omega_0/\omega_k}$, for Ω being the relative light-matter coupling constant, and ω_0 as the cavity frequency at $k = 0$. Further, $\hat{X}_n^\dagger$ ($\hat{a}_k^\dagger$) is the excitonic (photonic) creation operator at site n (wavevector k). Finally, $\hat{R}_{n,j}$ ($\hat{P}_{n,j}$) is the position (momentum) operator for the j-th phonon mode residing at nth site, where $r_n = na$ is the spatial location of the n-th site separated by the lattice constant a. Here we consider a 2D world and define $k = k_x\hat{\mathbf{x}} + k_y\hat{\mathbf{y}}$, noting that the cavity mirrors quantization direction lies in the y-direction and the exciton system is a 1-D line arranged in the x-direction. Here we consider the first cavity quantization along the y direction, thus $k_y = 2\pi/Ly$ for Ly being the cavity mirrors spacing. Since, we take the exciton system to be a single layer of material in the center of the cavity, we have $\sin(k_y\cdot r_n) = 1$. We now define a polaritonic Hamiltonian $\hat{H}_{\rm pl}(\mathbf{R}) = \hat{H}_{\rm LM} - \sum_n \hat{P}_{n,j}^2/2 - \omega^2 R_{n,j}^2/2$, expressed as

$$\begin{aligned}\hat{H}_{\rm pl} = &\sum_{n,j}\gamma_j \hat{X}_n^\dagger \hat{X}_n \hat{R}_{n,j} + \sum_n \hat{X}_n^\dagger \hat{X}_n \varepsilon_0 + \tau \sum_n (\hat{X}_n^\dagger \hat{X}_{n+1} + \hat{X}_{n+1}^\dagger \hat{X}_n) \\ &+ \sum_k \hat{a}_k^\dagger \hat{a}_k \omega_c(k) + \sum_{n,k}\frac{\Omega_k}{\sqrt{N}}(\hat{a}_k^\dagger \hat{X}_n e^{-ik_x\cdot r_n} + \hat{a}_k \hat{X}_n^\dagger e^{ik_x\cdot r_n}).\end{aligned} \tag{S2}$$

By imposing the classical-path approximation [4, 5, 6], we write the approximate classical force acting on the phonon degrees of freedoms within the mixed-quantum classical framework as $F_{n,j}(t) = \ddot{R}_{n,j}(t) = -\langle\Psi(t)|d\hat{H}_{\rm LM}(\mathbf{R})/dR_{n,j}|\Psi(t)\rangle$, where we treat the nuclear degrees of freedom classically, $\{\hat{R}_{n,j}, \hat{P}_{n,j}\} \to \{R_{n,j}, P_{n,j}\}$. The time dependence of $R_{n,j}$ is described as a harmonic oscillator, $R_{n,j}(t) = R_{n,j}(0)\cos(\omega_j t) + \dfrac{P_{n,j}(0)}{\omega_j}\sin(\omega_j t)$, where $R_{n,j}(0)$ and $P_{n,j}(0)$ are initial values of the nuclear position and momentum sampled from the Wigner distribution. Due to the classical-path approximation, the dynamics of the electronic-photonic sub-system can be described under the time-dependent polaritonic Hamiltonian as

$$\begin{aligned}\hat{H}_{\rm pl}(t) = &\sum_{n,j}\gamma_j \hat{X}_n^\dagger \hat{X}_n \Big(R_{n,j}(0)\cos(\omega_j t) + \frac{P_{n,j}(0)}{\omega_j}\sin(\omega_j t)\Big) + \sum_n \hat{X}_n^\dagger \hat{X}_n \varepsilon_0 + \tau \sum_n (\hat{X}_n^\dagger \hat{X}_{n+1} + \hat{X}_{n+1}^\dagger \hat{X}_n) \\ &+ \sum_k \hat{a}_k^\dagger \hat{a}_k \omega_c(k) + \sum_{n,k}\frac{\Omega_k}{\sqrt{N}}(\hat{a}_k^\dagger \hat{X}_n e^{-ik_x\cdot r_n} + \hat{a}_k \hat{X}_n^\dagger e^{ik_x\cdot r_n}).\end{aligned} \tag{S3}$$

We now expand this Hamiltonian into Sambe space, and define a set of complex numbers $Z_{n,j} = R_{n,j}(0)/2 + P_{n,j}(0)/2i\omega_j$ which describe the classical phonon field. Next, we separate the Hamiltonian into time-independent and time-dependent parts, $\hat{H}_{\rm pl}(t) = \hat{H}_0 + \hat{H}(t)$ where we define $\hat{H}_0$ and $\hat{H}(t)$ to be

$$\hat{H}_0 = \sum_n \hat{X}_n^\dagger \hat{X}_n \varepsilon_0 + \tau \sum_n (\hat{X}_n^\dagger \hat{X}_{n+1} + \hat{X}_{n+1}^\dagger \hat{X}_n) + \sum_k \hat{a}_k^\dagger \hat{a}_k \omega_c(k) + \sum_{n,k}\frac{\Omega_k}{\sqrt{N}}(\hat{a}_k^\dagger \hat{X}_n e^{-ik_x\cdot r_n} + \hat{a}_k \hat{X}_n^\dagger e^{ik_x\cdot r_n}) \tag{S4}$$

$$\hat{H}(t) = \sum_{n,j}\gamma_j \hat{X}_n^\dagger \hat{X}_n Z_{n,j} e^{i\omega_j t} + \sum_{n,j}\gamma_j \hat{X}_n^\dagger \hat{X}_n Z_{n,j}^* e^{-i\omega_j t}. \tag{S5}$$

If define the operators $\hat{P}_j$ and $\hat{P}_j{}^\dagger$ as $\hat{P}_j = \sum_n \gamma_j \hat{X}_n^\dagger \hat{X}_n Z_{n,j}$ and $\hat{P}_j{}^\dagger = \sum_n \gamma_j \hat{X}_n^\dagger \hat{X}_n Z_{n,j}^*$, the polariton Hamiltonian, $\hat{H}_{\rm pl}(t)$ resembles a standard Floquet Hamiltonian, where $\hat{H}_{\rm pl}(t) = \hat{H}_0 + \sum_j(\hat{P}_j e^{i\omega_j t} + \hat{P}_j{}^\dagger e^{-i\omega_j t})$ [7, 8]. Finally, we quantize the phonon field by following the standard Floquet theory [9, 10, 11].

S1.2 Polaron Transformation of the Phonon Field

In the following section, we rewrite $\hat{H}_{\rm pl}(t)$ in the Sambe (extended) space by introducing the phonon excitation operator $\hat{B}_j^\dagger$, which is a bosonic operator act on the Floquet block, $|m_j\rangle$, as follows

$$\hat{B}_j^\dagger |m_j\rangle = \sqrt{m_j+1}\, |m_j+1\rangle\,, \quad \hat{B}_j |m_j\rangle = \sqrt{m_j}\, |m_j-1\rangle\,. \tag{S6}$$

The Sambe space contains infinite Floquet blocks, but using a truncated Sambe space of Floquet blocks in our system guarantees the convergence of the results. Therefore, labeling the truncation value by F_j for each phonon mode, each Floquet block within this truncated finite space can be labeled by $m_j \in \{-F_j, -F_j+1, \ldots, F_j\}$, so the truncated Sambe space size is $\sum_j (2F_j+1)$. In our derivation, we require the Sambe truncated space to be shifted to a large Floquet block, where $|m_j\rangle \to |M_j + m_j\rangle$. Eventually, since we use the limit of $M_j \to \infty$, we use same shift $M_j = M$ for all of the phonon modes. Within this truncated space, the excitonic and photonic extended basis set $\{|\alpha\rangle, |\beta\rangle\}$ is given by

$$\{|\alpha\rangle, |\beta\rangle\} \;\in\; \Big\{ \hat{X}_n^\dagger \prod_j \frac{(\hat{B}_j^\dagger)^{M+m_j}}{\sqrt{(M+m_j)!}} |\bar{0}\rangle\,,\; \hat{a}_k^\dagger \prod_j \frac{(\hat{B}_j^\dagger)^{M+m_j}}{\sqrt{(M+m_j)!}} |\bar{0}\rangle \Big\}, \tag{S7}$$

where $|\bar{0}\rangle$ is the joint vacuum of excitons, photons, and phonons. In this basis the time-periodic Hamiltonian maps to a time-independent Floquet operator via

$$\hat{H}_{\rm pl}(t) \;\mapsto\; \hat{H}_F \;=\; \lim_{M\to\infty} \sum_{\alpha,\beta} |\beta\rangle \langle\beta|\, \hat{\mathcal{H}}_F\, |\alpha\rangle \langle\alpha|\,, \tag{S8}$$

Then the effective Hamiltonian can be written as

$$\begin{aligned}
\hat{\mathcal{H}}_{\rm F} &= \sum_n \varepsilon_0 \hat{X}_n^\dagger \hat{X}_n + \sum_j (\hat{B}_j^\dagger \hat{B}_j - M)\omega_j + \tau \sum_n (\hat{X}_n^\dagger \hat{X}_{n+1} + \hat{X}_{n+1}^\dagger \hat{X}_n) + \sum_j \frac{\gamma_j}{\sqrt{M}} \sum_n (Z_{n,j}\hat{B}_j + Z_{n,j}^* \hat{B}_j{}^\dagger)\hat{X}_n^\dagger \hat{X}_n \\
&\quad + \sum_k \hat{a}_k^\dagger \hat{a}_k \omega_c(k) + \sum_{n,k} \frac{\Omega_k}{\sqrt{N}} (\hat{a}_k^\dagger \hat{X}_n e^{-ik_x\cdot r_n} + \hat{a}_k \hat{X}_n^\dagger e^{ik_x\cdot r_n}) \\
&\approx \sum_n \varepsilon_0 \hat{X}_n^\dagger \hat{X}_n + \sum_j \omega_j \left(\hat{B}_j^\dagger + \hat{\alpha}_j^\dagger\right)\left(\hat{B}_j + \hat{\alpha}_j\right) + \tau \sum_n (\hat{X}_n^\dagger \hat{X}_{n+1} + \hat{X}_{n+1}^\dagger \hat{X}_n) \\
&\quad + \sum_k \hat{a}_k^\dagger \hat{a}_k \omega_c(k) + \sum_{n,k} \frac{\Omega_k}{\sqrt{N}} (\hat{a}_k^\dagger \hat{X}_n e^{-ik_x\cdot r_n} + \hat{a}_k \hat{X}_n^\dagger e^{ik_x\cdot r_n}) - \sum_j M\omega_j,
\end{aligned} \tag{S9}$$

where we have defined the operator $\hat{\alpha}_j^\dagger = \sum_n \hat{\alpha}_{n,j}^\dagger = \sum_n \frac{\gamma_j}{\omega_j\sqrt{M}} Z_{n,j} \hat{X}_n^\dagger \hat{X}_n$, where $\sqrt{M}$ was added to scale down the exciton-phonon coupling γ_j when $M \to \infty$. Next, we perform a polaron transformation to displace the phonon field using the unitary operator $\hat{U}_{\rm D}$, which is defined as

$$\hat{U}_{\rm D} = \prod_{n,j} \hat{U}_{{\rm D},nj} = \prod_{n,j} e^{\hat{\alpha}_{n,j}\hat{B}_j{}^\dagger - \hat{\alpha}_{n,j}^\dagger \hat{B}_j}. \tag{S10}$$

Following straightforward algebraic transformations under $\hat{U}_{\rm D}$ we get

$$\hat{\mathcal{H}}_{\rm F}' = \hat{U}_{\rm D}\hat{\mathcal{H}}_{\rm F}\hat{U}_{\rm D}^\dagger = \sum_n \varepsilon_0 \hat{X}_n^\dagger \hat{X}_n + \sum_j (\hat{B}_j{}^\dagger \hat{B}_j - M)\omega_j + \tau \sum_n \big(\hat{X}_n^\dagger \hat{X}_{n+1} \prod_j e^{-(\hat{\phi}_{n,j} - \hat{\phi}_{n+1,j})} + h.c.\big) \tag{S11}$$

$$+ \sum_k \hat{a}_k^\dagger \hat{a}_k \omega_c(k) + \sum_{n,k} \frac{\Omega_k}{\sqrt{N}} \prod_j \big(\hat{a}_k^\dagger \hat{X}_n e^{-ik_x\cdot r_n} e^{\hat{\phi}_{n,j}} + h.c\big). \tag{S12}$$

where we defined $\hat{\phi}_{n,j} = \Big[\frac{\gamma_j}{\omega_j\sqrt{M}} (Z_{n,j}^* \hat{B}_j{}^\dagger - Z_{n,j}\hat{B}_j)\Big]$.

S1.3 Analytical Expression of Overlaps

To express $\hat{\mathcal{H}}_{\rm F}$ in the basis $\{|\alpha\rangle, |\beta\rangle\}$, we compute the overlap between infinitely excited displaced Fock states of the jth phonon mode written as

$$\langle M+m_j'|\, e^{\hat{\phi}_{n,j}}\, |M+m_j\rangle = \lim_{M\to\infty} \left\langle M+m_j' \left| \exp\left[-\frac{\gamma_j (Z_{n,j}\hat{B}_j - Z_{n,j}^*\hat{B}_j^\dagger)}{\omega_j\sqrt{M}}\right] \right| M+m_j \right\rangle = Q_{m_j'm_j}^{[j]}(Z_{n,j}), \tag{S13}$$

where the analytical form of the function $Q^{[j]}_{m_j m'_j}(\tilde{Z})$ (with $\tilde{Z}$ as a complex number) is written as

$$Q^{[j]}_{m_j m'_j}(\tilde{Z}) = \lim_{M\to\infty}\left(\exp\Big[-\frac{\gamma_j^2|\tilde{Z}|^2}{2\omega_j^2 M}\Big]\sqrt{\frac{(M+\min\{m'_j,m_j\})!}{(M+\max\{m'_j,m_j\})!}}\begin{cases}\left(\frac{\gamma_j\tilde{Z}^*}{\omega_j\sqrt{M}}\right)^{m_j-m'_j} L^{(|m_j-m'_j|)}_{\min\{m_j,m'_j\}}\left((\frac{\gamma_j}{\omega_j\sqrt{M}})^2|\tilde{Z}|^2\right), & m'_j\le m_j\\ \left(-\frac{\gamma_j\tilde{Z}}{\omega_j\sqrt{M}}\right)^{m'_j-m_j} L^{(|m'_j-m_j|)}_{\min\{m_j,m'_j\}}\left((\frac{\gamma_j}{\omega_j\sqrt{M}})^2|\tilde{Z}|^2\right), & m'_j> m_j\end{cases}\right) \tag{S14}$$

In the following, we will also need the term $\mathcal{S}^{[j]}_{m_j} = \sum_n |Q^{[j]}_{m_j,0}(Z_{n,j})|^2 = \int \mathcal{W}_{n,j}|Q_{m_j0}(z)|^2\,dz$, where we converted summation to integration by assuming $Z_{n,j}$ to be represented by the continues variable z, and $\mathcal{W}_{n,j}$ is the Wigner quasi-probability density which is given by

$$\mathcal{W}_{n,j} = 2\tanh\left(\frac{\omega_j}{2k_BT}\right)\cdot\exp\left[-\tanh\left(\frac{\omega_j}{2k_BT}\right)\left(\omega_j R_j(0)^2+\frac{P_j(0)^2}{\omega_j}\right)\right] \tag{S15}$$

$$= 2\tanh\left(\frac{\beta\omega_j}{2}\right)\cdot\exp\left[-4\omega_j\tanh\left(\frac{\beta\omega_j}{2}\right)z^2\right] \tag{S16}$$

Using this, we can write

$$\mathcal{S}^{[j]}_{m_j} = \lim_{M\to\infty} 2\tanh\Big(\frac{\beta\omega_j}{2}\Big)\int_{-\infty}^{\infty} dz\frac{M!}{(M+m_j)!}\left(\frac{\gamma_j^2 z^2}{\omega_j^2 M}\right)^{m_j}\times\exp\left[-\Big(4\omega_j\tanh\Big(\frac{\beta\omega_j}{2}\Big)+\frac{z^2\gamma_j^4}{4\omega_j^4M^2}\Big)z^2\right]\left[L^{(m_j)}_M\Big(\frac{\gamma_j^2z^2}{\omega_j^2M}\Big)\right]^2.$$

In the main text, we numerically compute $\mathcal{S}^{[j]}_{m_j}$ by sampling $\{z\}$ from the Wigner distribution for each ω_j.

The splitting $\propto\sqrt{\mathcal{S}^{[0]}_m} = \sqrt{\sum_n\left[Q^{[0]}_{m0}(Z_n)\right]^2}$ is obtained accurately when including the fluctuations Z_n of all phonon modes in the system and cannot be estimated accurately using some expectation value of Z_n since $\sqrt{\mathcal{S}^{[0]}_m}\neq\sqrt{\left[Q^{[0]}_{m0}(\langle Z_n\rangle)\right]^2}$, where $\langle ...\rangle$ indicates phase space averaging.

S1.4 Polaron-Polariton Quasi-Band Picture in Reciprocal Space

Below we introduce the polaron-polariton quasi-band picture. First we consider a exciton-phonon-photon basis $\{|f_i\rangle\}$ which in term of the creation operators are given by

$$|f_i\rangle\in\begin{cases}\Big|n,\bigotimes_{j=1}^{N_b}(M+m_j),0\Big\rangle = \hat{X}^\dagger_n\prod_j\frac{(B^\dagger_j)^{M+m_j}}{\sqrt{(M+m_j)!}}|\bar{0}\rangle\\ \Big|0,\bigotimes_{j=1}^{N_b}M,1_k\Big\rangle = \hat{a}^\dagger_k\prod_j\frac{(B^\dagger_j)^M}{\sqrt{M!}}|\bar{0}\rangle\end{cases}\quad\text{and}\quad \mathbb{P}'_i = |f'_i\rangle\langle f'_i|\,. \tag{S17}$$

Where N_b is the number of phonon modes per site. Furthermore, we adapt the notation $|n,\vec{\mathbf{m}}\rangle\equiv\Big|n,\bigotimes_{j=1}^{N_b}(M+m_j),0\Big\rangle$, $|1_k\rangle\equiv\Big|0,\bigotimes_{j=1}^{N_b}M,1_k\Big\rangle$, and $\hat{\mathcal{H}}'_\mathrm{F} = \lim_{M\to\infty}\sum_{ij}\mathbb{P}'_j\hat{\mathcal{H}}_\mathrm{F}\mathbb{P}'_i$, where $\hat{\mathcal{H}}'_\mathrm{F}$ can be expressed as (we define $\Delta Z_{n,j} = Z_{n+1,j}-Z_{n,j}$).

$$\hat{\mathcal{H}}'_\mathrm{F} = \Bigg[\sum_k\omega_c(k)\big|1_k\big\rangle\big\langle 1_k\big| + \sum_{n,m,j}\big(\varepsilon_0+m_j\omega_j\big)\ \big|n,\vec{\mathbf{m}}\big\rangle\big\langle n,\vec{\mathbf{m}}\big| + \tau\sum_{m,m',n}\prod_j Q^{[j]}_{m_jm'_j}(\Delta Z_{n,j})\Big(\big|n,\vec{\mathbf{m}}\big\rangle\big\langle n+1,\vec{\mathbf{m}}'\big| + h.c.\Big)$$

$$+\sum_{m,n,k}\prod_j\frac{\Omega_k}{\sqrt{N}}\Big(Q^{[j]}_{0m_j}(Z_{n,j})e^{-ik\cdot r_n}\big|n,\vec{\mathbf{m}}\big\rangle\big\langle 1_k\big| + h.c.\Big)\Bigg].$$

We will now define effective reciprocal space exciton states $\big|k,\vec{\mathbf{m}}\big\rangle = \hat{Y}^\dagger_{k,\vec{\mathbf{m}}}|\bar{0}\rangle$ that are approximately (see next section) orthogonal to each other

$$\big|k,\vec{\mathbf{m}}\big\rangle = \sum_n\prod_j\frac{Q^{[j]}_{0m_j}(Z_n)}{\sqrt{\mathcal{S}^{[j]}_{m_j}}}e^{-ik\cdot r_n}\big|n,\vec{\mathbf{m}}\big\rangle\equiv\hat{Y}^\dagger_{k,\vec{\mathbf{m}}}|\bar{0}\rangle. \tag{S18}$$

Next we replace the hopping term with the expectation value $\big\langle k,\vec{\mathbf{m}}\big|\tau\sum_{m,m',n}Q^{[j]}_{m_jm'_j}(\Delta Z_n)\Big(\big|n,\vec{\mathbf{m}}\big\rangle\big\langle n+1,\vec{\mathbf{m}}'\big|+h.c\Big)\big|k,\vec{\mathbf{m}}\big\rangle\approx 2\tau\sum_n\prod_j Q^{[j]}_{00}(Z_{n+1}-Z_n)\cdot Q^{[j]}_{0m_j}(Z_n)\cdot Q^{[j]}_{0m_j}(Z_{n+1,j})/\mathcal{S}^{[j]}_{m_j} = 2\tau\cdot\xi_0$. With this, we write the final form of the polaron-polariton Hamiltonian in the Sambe space as

$$\hat{H}'_\mathrm{F}\approx\sum_k\Bigg[\hat{a}^\dagger_k\hat{a}_k\omega_c(k)+\sum_{m,j}(\varepsilon_0+m_j\omega_j)\hat{Y}^\dagger_{k,\vec{\mathbf{m}}}\hat{Y}_{k,\vec{\mathbf{m}}}+\sum_m 2\tau\cdot\xi_0\hat{Y}^\dagger_{k,\vec{\mathbf{m}}}\hat{Y}_{k,\vec{\mathbf{m}}}$$

$$+\sum_m\prod_j\sqrt{\frac{\mathcal{S}^{[j]}_{m_j}}{N}}\Omega_k\Big(\hat{Y}^\dagger_{k,\vec{\mathbf{m}}}\hat{a}_k+\hat{a}^\dagger_k\hat{Y}_{k,\vec{\mathbf{m}}}\Big)\Bigg] = \sum_k\hat{\mathcal{H}}_k.$$

Note that there exists an infinite number of energetically relevant $\hat{Y}^{\dagger}_{k,\vec{\mathbf{m}}}$ operators that could couple to the photonic operators $\hat{a}_k$. However only a few finite number of energetically relevant $\hat{Y}^{\dagger}_{k,\vec{\mathbf{m}}}$ operators have non-negligible couplings $\prod_j \sqrt{\frac{\mathcal{S}^{[j]}_{m_j}}{N}}\Omega_k$. In our multimode calculations, we ignore any $\hat{Y}^{\dagger}_{k,\vec{\mathbf{m}}}$ for which $\prod_j (\mathcal{S}^{[j]}_{m_j}/N) \leq 0.000025$.

S1.5 Orthogonality of the Effective Reciprocal Excitonic states

Consider the effective reciprocal exciton states including multiple phonon modes:

$$\big|k,\vec{\mathbf{m}}\big\rangle = \sum_n \prod_j \frac{Q^{[j]}_{0m_j}(Z_{h,j})}{\sqrt{\mathcal{S}^{[j]}_{m_j}}} e^{-ik\cdot r_n}\big|n,\vec{\mathbf{m}}\big\rangle \equiv \sum_h^{N_\mathrm{C}} \sum_i^{N/N_\mathrm{C}} \prod_j \frac{Q^{[j]}_{0m_j}(Z_{(hN_C+i),j})}{\sqrt{\mathcal{S}^{[j]}_{m_j}}} e^{-ik\cdot r_{h\cdot N_\mathrm{C}+i}}\big|(hN_\mathrm{C}+i),\vec{\mathbf{m}}\big\rangle. \tag{S19}$$

where in the right-hand side we have introduced supercells of size N_C that are indexed by h. Within each of these supercells, each unit cell is indexed by i. Such that we have the mapping of the state $\big|n,m\big\rangle \mapsto \big|(hN_\mathrm{C}+i),\vec{\mathbf{m}}\big\rangle$. The above equation can be approximately written as

$$\big|k,\vec{\mathbf{m}}\big\rangle \approx \sum_h \sum_i \prod_j \frac{Q^{[j]}_{0m_j}(Z_{h,j})}{\sqrt{\mathcal{S}^{[j]}_{m_j}}} e^{-ik\cdot r_{hN_\mathrm{C}}}\big|(hN_\mathrm{C}+i),\vec{\mathbf{m}}\big\rangle \equiv \sum_h e^{-ik\cdot r'_h}\big|\mathrm{C}_h,\vec{\mathbf{m}}\big\rangle. \tag{S20}$$

where $r_{hN_\mathrm{C}} = r'_h$ and $\big|\mathrm{C}_h,\vec{\mathbf{m}}\big\rangle = \sum_i \prod_j \frac{Q^{[j]}_{0m_j}(Z_{h,j})}{\sqrt{\mathcal{S}^{[j]}_{m_j}}}\big|(hN_\mathrm{C}+i),\vec{\mathbf{m}}\big\rangle$. The approximate form on the right-hand side is arrived at by assuming that (a) the length of the supercell containing N_C items is much smaller than the wavelengths of the radiation, i.e. $e^{ik\cdot(N_\mathrm{C}a)} \approx 1$ such that the box size is much smaller than the wavelength of the radiation, and (b) each box contains a statistically sufficient number of sites with disorders sampled from a Wigner distribution such that for any given site i, the summation of the product of overlap function $\prod_j \frac{Q^{[j]}_{0m_j}(Z_{h,j})}{\sqrt{\mathcal{S}^{[j]}_{m_j}}}$ remains equivalent across boxes. Therefore, it follows that the effective reciprocal exciton states $\{|k,\vec{\mathbf{m}}\rangle\}$ are approximately orthogonal

$$\big\langle k',\vec{\mathbf{m}}\big|k,\vec{\mathbf{m}}\big\rangle \approx \delta_{kk'}. \tag{S21}$$

S1.6 Generalization of Phonon-Polariton Theory to Incorporate Angular Molecular Disorder

Our theory can be easily extended to include angular disorder, which would be apt for molecular polaritons. In the presence of the angular disorder, the total light-matter Hamiltonian is written as

$$\begin{aligned}\hat{H}_\mathrm{LM} = &\sum_n \hat{X}^{\dagger}_n\hat{X}_n\varepsilon_0 + \sum_k \hat{a}^{\dagger}_k\hat{a}_k\omega_c(k) + \sum_{n,j}\frac{\hat{P}^2_{n,j}}{2} + \frac{1}{2}\omega_j^2\hat{R}^2_{n,j} + \tau\sum_n(\hat{X}^{\dagger}_n\hat{X}_{n+1} + \hat{X}^{\dagger}_{n+1}\hat{X}_n) + \sum_{n,j}\gamma_j\hat{X}^{\dagger}_n\hat{X}_n\hat{R}_{n,j}\\ &+\sum_{n,k}\frac{\Omega_k\theta_n}{\sqrt{N}}\left[\hat{a}^{\dagger}_k\hat{X}_n e^{-ik\cdot r_n} + \hat{a}_k\hat{X}^{\dagger}_n e^{ik\cdot r_n}\right],\end{aligned} \tag{S22}$$

where θ_n accounts for the angular disorder. Following the same steps as in the main-text, one arrives at at the following light-matter Hamiltonian in the Sambe space when invoking the phonon field description,

$$\begin{aligned}\hat{H}'_\mathrm{F} &= \lim_{M\to\infty}\sum_{ij\in\mathcal{S}}\mathbb{P}_j\hat{\mathcal{H}}'_\mathrm{F}\mathbb{P}_i = \lim_{M\to\infty}\sum_{ij\in\mathcal{S}}\mathbb{P}_j(\hat{U}^{\dagger}_D\hat{\mathcal{H}}_\mathrm{F}\hat{U}_D)\mathbb{P}_i \\ &= \Bigg[\sum_k\omega_c(k)\big|1_k\big\rangle\big\langle 1_k\big| + \sum_{n,\vec{\mathbf{m}},j}\left(\varepsilon_0 + m_j\omega_j\right)\,\big|n,\vec{\mathbf{m}}\big\rangle\big\langle n,\vec{\mathbf{m}}\big| + \tau\sum_{m,m',n}\prod_j Q^{[j]}_{m_jm'_j}(\Delta Z_{n,j})\Big(\big|n,\vec{\mathbf{m}}\big\rangle\big\langle n+1,\vec{\mathbf{m}}'\big| + h.c.\Big)\\ &+\sum_{m',n,k}\frac{\Omega_k\theta_n}{\sqrt{N}}\prod_j\frac{Q^{[j]}_{0m_j}(Z_{n,j})}{\sqrt{\mathcal{S}^{[j]}_{m_j}}}e^{-ik\cdot r_n}\big|n,\vec{\mathbf{m}}\big\rangle\big\langle 1_k\big| + h.c.\Big)\Bigg].\end{aligned} \tag{S23}$$

Under such circumstances, we will define a slightly modified form of the effective reciprocal exciton state as

$$\big|k,\vec{\mathbf{m}}\big\rangle = \sum_n\prod_j\frac{Q^{[j]}_{0m_j}(Z_{n,j})}{\sqrt{\mathcal{S}^{[j]}_{m_j}}}\theta_n e^{-ik\cdot r_n}\big|n,\vec{\mathbf{m}}\big\rangle \tag{S24}$$

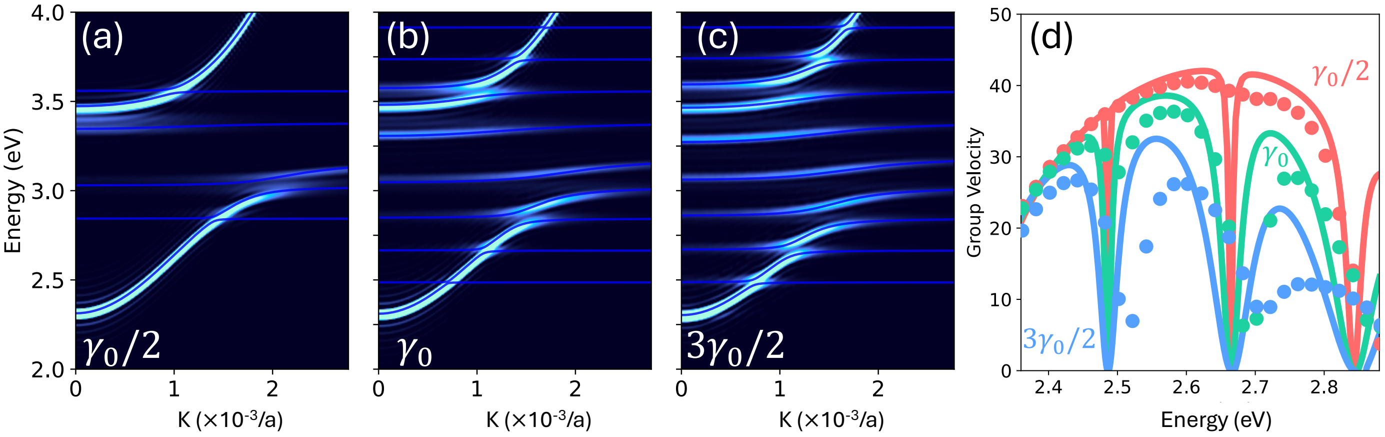

Figure S1: Exciton-polariton band structure from mixed Quantum-Classical Simulation and Analytical Theory. (a) With phonon coupling $\gamma_0/2$, (b) With phonon coupling γ_0, (c) With phonon coupling $3\gamma_0/2$, where γ_0 is the phonon coupling strength. The parameter $\gamma_0 = 0.0159$ eV. (d) Group velocities extracted from quantum dynamical simulations (filled circles) compared to the predictions of the analytical model (solid lines) introduced in this work. Here, we use $\Omega = 3900$ cm^{-1}, $N = 30001$, $\tau = 200$ cm^{-1}, $\omega = 1440$ cm^{-1} $\omega_c(0) = 2.58$ eV, and $\varepsilon_0 = 3.2$ eV.

where $\mathcal{S}^{[j]}_{m_j} = \sum_{n_j} |Q^{[j]}_{0m_j}(Z_{n,j})|^2$. Following the same steps as in Sec. S1.5, we can show that these exciton states $\{|k, \vec{\mathbf{m}}\rangle\}$ are orthogonal.

S2 Initialization of the Exciton-Polariton Wavefunction via Monte Carlo Sampling

We initialize the exciton-polariton wavefunction as a linear combination of lower-polariton states

$$|\Psi(0)\rangle = \sum_{k\in\mathcal{E}_{\text{init}}} c_k \hat{P}^\dagger_{k,-}|\bar{0}\rangle \tag{S25}$$

where $\mathcal{E}_{\text{init}}$ defines a subspace corresponding to the lower polariton states of energy $E_{k,-}$ that lie within an energy window $E_{\text{init}} - \Delta/2 < E_{k,-} < E_{\text{init}} + \Delta/2$. Here, the lower polariton operator $\hat{P}^\dagger_{k,-}$ is obtained by diagonalizing the exciton-polariton Hamiltonian (in the absence of the phonons)

$$\begin{aligned}\hat{H}_{\text{EP}} &= \sum_n \hat{X}^\dagger_n \hat{X}_n \varepsilon_0 + \sum_k \hat{a}^\dagger_k \hat{a}_k \omega_c(k) + \tau \sum_n (\hat{X}^\dagger_n \hat{X}_{n+1} + \hat{X}^\dagger_{n+1}\hat{X}_n) + \sum_{n,k} \frac{\Omega_k}{\sqrt{N}}\left[\hat{a}^\dagger_k \hat{X}_n e^{-ik\cdot r_n} + \hat{a}_k \hat{X}^\dagger_n e^{ik\cdot r_n}\right] \\ &= \sum_k \hat{X}^\dagger_k \hat{X}_k \varepsilon_k + \sum_k \hat{a}^\dagger_k \hat{a}_k \omega_c(k) + \sum_k \Omega_k \left[\hat{a}^\dagger_k \hat{X}_k + \hat{a}_k \hat{X}^\dagger_k\right] = \sum_k \hat{P}^\dagger_{k,+}\hat{P}_{k,+}E_+(k) + \sum_k \hat{P}^\dagger_{k,-}\hat{P}_{k,-}E_-(k)\end{aligned} \tag{S26}$$

where $E_\pm(k)$ the polaritonic energies. We select the coefficients c_k by minimizing

$$\sum_n |\langle\bar{0}|\hat{X}_n|\Psi(0)\rangle|^2 (n - N/2) \tag{S27}$$

such that $|\Psi(0)\rangle$ represents an energetically as well as a spatially localized wavefunction at $t = 0$.

S3 Additional Results

Fig. S1 presents our numerical and analytical results in the presence of a hopping term $\tau = 200$ cm^{-1}. Fig. S1. Fig. S2 presents our numerical and analytical results at intermediate phonon frequency $\omega = 720$ cm^{-1}. Fig. S2. Overall, in both cases, our theoretical predictions match the numerical results reasonably well, as expected.

S3.1 Comparing to Exact Diagonalization

We performed exact calculations for the exciton-polariton model on a reduced system size. Fig. S3 and S4 correspond to the one-site and two-site systems, respectively, at $T = 0$ K. In both cases, the system was initialized with an excitation on the first photonic site and evolved for approximately ~ 0.5 ps. The spectral densities computed using MFE show strong

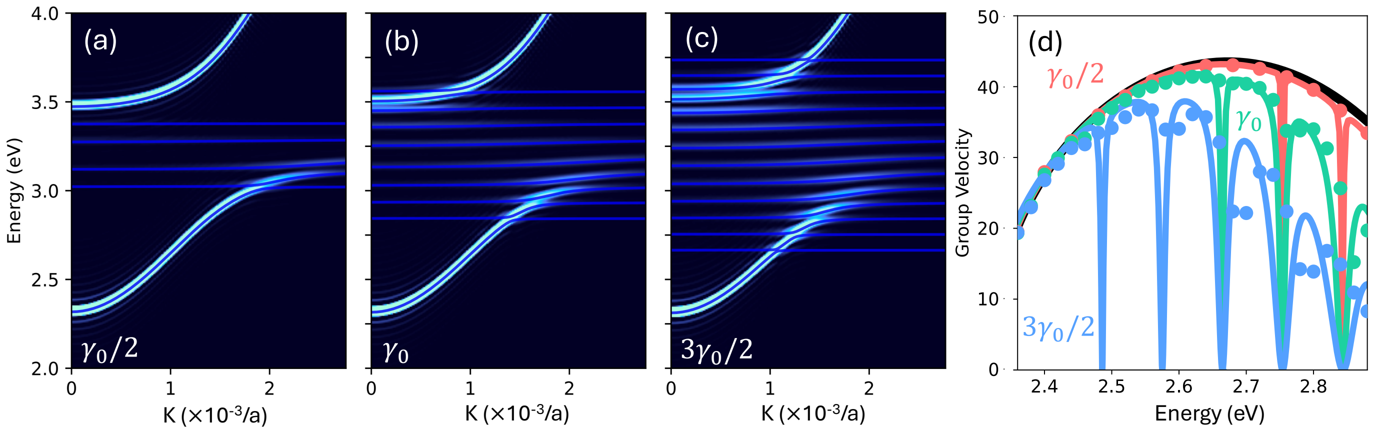

Figure S2: Exciton-polariton band structure from mixed Quantum-Classical Simulation and Analytical Theory. (a) With phonon coupling $\gamma_0/2$, (b) With phonon coupling γ_0, (c) With phonon coupling $3\gamma_0/2$, where γ_0 is the phonon coupling strength. The parameter $\gamma_0 = 0.00795$ eV. (d) Group velocities extracted from quantum dynamical simulations (filled circles) compared to the predictions of the analytical model (solid lines) introduced in this work. All computations used a phonon frequency of 720 cm^{-1}. Further, we use $\Omega = 3900$ cm^{-1}, $N = 40001$, $\tau = 0$, $\omega_c(0) = 2.58$ eV, and $\varepsilon_0 = 3.2$ eV.

agreement with the exact results, where the phonon Hilbert space was truncated to include up to the fifth excited phonon state. Evolution of the correlation function with respect to time (Fig S3 and S4, Panel C) also matches closely between the two methods. Fig . S5 presents the angle-resolved absorption spectra for a seven-site system (comprising 7 excitonic sites, 7 photonic modes, and 7 phonon modes). Clear signatures of vibrational band splitting are observed using both methods: Exact (panels a and c) and MFE (panels b and d). While MFE slightly underestimates the spectral intensities, it accurately reproduces the peak positions. This demonstrates the reliability and applicability of the MFE approach for modeling exciton-polariton dynamics in this system.

That said, there are small deviation between the MFE and the exact approach. Specifically, note that spectral line-widths are larger in MFE than in exact and the heights of the peaks (intensities) predicted in MFE can be off by up-to $\sim 20\%$ (for secondary peaks) for the range of parameters explored in these few-site models at $T = 0$ K. Note that, MFE is also expected to perform much better at room-temperature (which is the focus of our work), as we have shown in our recent work [12].

S3.2 Comparing to Quantum Perturbative Theory

To ensure the applicability of the MFE method, we compare it to a Green's function-based perturbative approach discussed in [13]. For the relatively weak phonon couplings used in those cases, our numerical MFE simulations, the analytical approach developed in this work, and the quantum mechanical perturbation theory (QM-PT) produce identical results. As expected, however, QM-PT fails to capture vibronic splitting features (e.g., the dip in Fig. S6), which become evident in regimes with stronger coupling.

Fig. S6 further illustrates this comparison by evaluating group velocities for two different cases. Fig. S6(a) presents a low phonon frequency scenario with $\omega_0 = 360\,\text{cm}^{-1}$ and exciton-phonon coupling strength $\gamma = 7.3 \times 10^{-5}$ a.u. in a system of $N = 30001$. Here, the group velocities from MFE simulations (red circles), perturbative theory (dashed red line), and our analytical model (solid red line) all show excellent agreement, confirming the validity of perturbative assumptions under weak coupling. Fig. S6(b) investigates a high phonon frequency case with $\omega_0 = 1440\,\text{cm}^{-1}$ and a stronger coupling $\gamma = 5.85 \times 10^{-4}$ a.u. in a larger system with $N = 40001$. In this regime, the QM-PT approach breaks down, failing to capture the modified group velocity structure due to significant vibronic interactions. Our analytical theory, however, remains accurate and closely follows the MFE simulation results. This figure emphasizes that while QM-PT can be sufficient in weak coupling regimes, our analytical method retains predictive accuracy across a broader range of parameters.

S3.3 Absorption Spectra of Multimode Exciton-Polariton Systems

Fig. S7 and Fig. S8 present a comprehensive analysis of the exciton-polariton dispersion and group velocity behavior in multimode systems coupled to structured phonon environments. Both simulation results from Quantum Dynamics via Mean Field Ehrenfest (MFE) and analytical theory predictions are compared across systems with varying phonon spectral complexity. Fig. S7 presents a comparative study of angle-resolved absorption spectra and group velocities for two-mode exciton-polariton systems under varying phonon spectral densities. The spectral densities in the first coloumn correspond to different phonon coupling configurations, where the coupling strength γ_0 associated with the low-frequency phonon mode $\omega_0 = 300\,\text{cm}^{-1}$ is systematically varied across rows, while the high-frequency mode $\omega_1 = 1400\,\text{cm}^{-1}$ remains fixed at $\gamma_1 = 2.8 \times 10^{-4}$ a.u. Note that the inclusion of low-frequency phonon modes effectively resembles the same effects as including a static disorder.

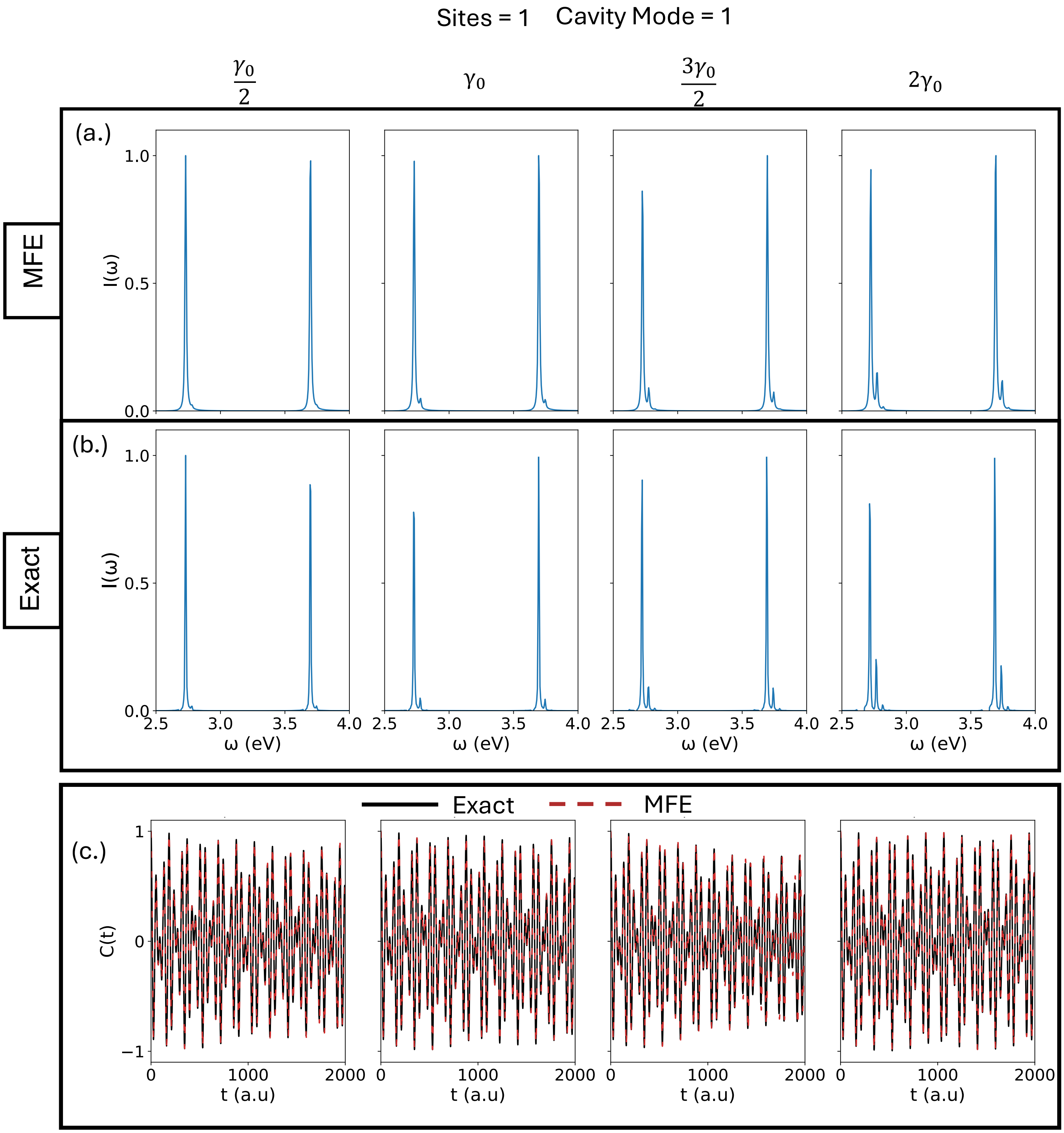

Figure S3: Polariton spectra with $N = 1$ calculated using the mean field Ehrenfest (MFE) method (top row) and the exact diagonalization (bottom row) approach for different phonon coupling strengths, using a reference coupling constant of $\gamma_0 = 3.8898 \times 10^{-5}$ a.u. and a phonon frequency of $\omega = 400$ cm^{-1}.

The second column shows the angle-resolved spectra obtained from MFE simulations, while the third column presents the corresponding results from the analytical theory. In all cases, there is excellent agreement between simulation and theory, both in the overall spectral structure and in the appearance of vibrational sidebands induced by phonon coupling. As γ_0 increases from 0 to 1.389×10^{-4} a.u., additional spectral features emerge due to stronger vibronic mixing, most notably in the bottom row, where multiple sidebands are visible.

Fig. S7(d),(h),(l) compares the group velocities extracted from MFE simulations (red dots) with those predicted by the theory (solid red lines). Notably, even with increased vibronic complexity (as in Fig. S7(l)), the theoretical predictions closely follow the simulated values, demonstrating the predictive accuracy of the analytical model. Our results further demonstrate that the presence of a low-frequency phonon mode can significantly suppress the vibronic structure associated with a high-frequency phonon mode, as observed in conventional molecular spectra.

Fig. S8 extends this comparison across systems with an increasing number of phonon modes: one Fig. S8(a–d), two Fig. S8(e–h), three Fig. S8(i–l), and five Fig. S8(m–p). Fig. S8(a),(e),(i),(m) show the cumulative phonon spectral densities, with the phonon frequencies $\omega_0 = 300\,\mathrm{cm}^{-1}$, $\omega_1 = 520\,\mathrm{cm}^{-1}$, $\omega_2 = 860\,\mathrm{cm}^{-1}$, $\omega_3 = 1110\,\mathrm{cm}^{-1}$, and $\omega_4 = 1400\,\mathrm{cm}^{-1}$ added in ascending order. The central panels in Fig. S8 compare the simulated (left) and theoretical (right) spectra, and Fig. S8(b–g), Fig. S8(f–g), Fig. S8(j–k), and Fig. S8(n–o) demonstrating how additional phonon modes broaden the vibronic structure and increase polariton dispersion. Fig. S8(d),(h),(l),(p) again confirm that group velocity predictions remain consistent between the two approaches, even as the number of phonon interactions increases. The coupling constants used are $\gamma_0 = 1.85 \times 10^{-5}$ a.u., $\gamma_1 = 4.23 \times 10^{-5}$ a.u., $\gamma_2 = 8.99 \times 10^{-5}$ a.u., $\gamma_3 = 1.32 \times 10^{-4}$ a.u., and $\gamma_4 = 1.86 \times 10^{-4}$ a.u.. Overall, Fig. S7 and Fig. S8 confirm the validity and precision of the proposed analytical theory in reproducing not only energy dispersions but also dynamical features like group velocities, across a wide range of spectral densities and phonon couplings.

S3.4 Anharmonic Considerations

We perform the spectral calculations identical to those in the main text, but with phonons propagating on anharmonic potentials modeled using the morse potential:

$$V_{\mathrm{anh}}(R) = D_e(1 - e^{-aR})^2 \tag{S28}$$

where $D_e = \dfrac{\omega}{4\chi_E}$ is the dissociation energy and $a = \sqrt{2\omega\chi_E}$ relates to the corresponding Harmonic frequency ω, both of which are related to a anharmonicity factor χ_E. Therefore the full exciton-polariton light-matter Hamiltonian simulated here, is written as (see Eq. 1 in the main-text)

$$\hat{H}_{\mathrm{LM}} = \hat{H}_{\mathrm{EP}} + \gamma \sum_n \hat{X}_n^\dagger \hat{X}_n \hat{R}_n + \sum_n \frac{\hat{P}_n^2}{2} + V_{\mathrm{anh}}(\hat{R}_n). \tag{S29}$$

We use $\gamma = 2.923 \times 10^{-4} a.u.$, $\omega = 1440$ cm^{-1} with the rest of the parameters identical to Fig. 1 in the main-text. Typical organic molecules, such as for the ring breathing mode at ~ 1400 cm^{-1} in polycyclic aromatic hydrocarbons (anthracene, hexacene, pentacene etc. and their derivatives) which exhibit vibronic structure in polaritonic dispersion [14, 15, 16, 17, 18], have $\chi_E \approx 0.01$ [19].

In Fig. S11 we consider three parameter regimes, $\chi_E = (0, 0.005, 0.01)$ with $\chi_E = 0$ corresponding to the Harmonic limit. Fig. S11 illustrates that increasing anharmonicity for the range of parameters chosen here does not significantly alter the polaritonic dispersion. That said larger anharmonicity, other forms of anharmonicity (e.g. quartic anharmonicity [20, 21]), and mode-mode coupling may impact the dynamics and spectra of the exciton-polaritons even at short time scales, which will be the focus of our future work.

S3.5 Temperature Considerations

We perform dynamics at various temperatures, for the case of $\omega_0 = 360$ cm^{-1}, exciton-phonon coupling strength $\gamma = 7.3 \times 10^{-5}$ a.u., and compare to the propagation without phonon coupling, to examine the scaling relation of our model with temperature. Similar to a recent experimental work, [14] we find qualitative agreement (See [14] Fig. 3c, that at increased temperatures, the Group velocity is hindered, similar to the effects as exciton-phonon coupling increases, which we present in Fig. S9. We compute the percent group velocity renormalization as

$$\%V_g(T) = \left[-\frac{(V_{g,0} - V_g(T))}{V_{g,0}} \right] \cdot 100\% \tag{S30}$$

where $V_{g,0}$ is the group velocity without phonon coupling, which is not temperature dependent. We find that there is a non-linear decrease in the group velocity as temperature increases, very similar to that seen in the prior mentioned experiment [14]. Note that prior theoretical works [13, 22] fail to capture this non-linear scaling between group velocity and temperature.

Lattice Spacing (a)	Nuclear Time Steps	Electronic Time Step	Refractive Index (η)
22.68	20	0.5	2.4

Table S1: Physical Parameters for the System Used to generate Fig 1, (in atomic units).

S3.6 Finite Cavity Lifetime

In the main text, we assume a lossless cavity, however here we introduce a finite cavity lifetime (a lossy cavity) via a leaking parameter Γ, which is experimentally relevant for polariton transport, and shown to exhibit significantly different dynamics (non-linear Mean-Squared Displacement), emphasized in Tichauer et al's recent work [23]. We implement the finite cavity lifetime as

$$\hat{H}_{\text{Loss}} = \sum_k -i\Gamma(a_k^\dagger a_k) \tag{S31}$$

where population decays from the photonic component of the system. We provide the modification to the polariton dispersion (see Fig. S12) and group velocity at when considering cavity loss up to $\Gamma = 5$ meV (see Fig. S13). We find that the observed polartion dispersion exhibits a line-broadening of the band structure, however the central positions of the bands are unaltered, where our theory is still applicable. Additionally, we observe that the group velocity is minimally altered up to $\Gamma = 5$ meV, where we compute the propagation rate for the furthest 2% of the respective population (normalized in the case of the cavity). We note however that at shorter cavity lifetimes (more lossy cavities) and longer propagation times (beyond 250 fs), the effects of cavity loss may drastically alter the propagation of polaritons in cavity systems turning a ballistic propagation into diffusive motion. As group velocity is only defined for a coherent, ballistic trajectory, our theory, which at present only defines the initial coherent motion is not applicable, which we plan to extend upon in future works.

S4 Parameters Used For Simulation

In the main text, we have employed semiclassical approaches, specifically MFE, to derive the optical band structure for varying levels of phonon coupling, as shown in FIG. 1. These simulations use $\Omega = 3900$ cm^{-1}, $N = 40001$, $\tau = 0$, $\omega_c(0) = 2.58$ eV, and $\varepsilon_0 = 3.2$ eV, unless otherwise stated. The general parameters used for all the numerical simulations are tabulated in Table 1 of SI. Due to the macroscopic size of our system, we find that as few as 10 trajectories are sufficient to obtain converged results for the photonic spectra and group velocities presented in this work. We use 30 trajectories in the main text to obtain the group velocities presented in Fig. 2.

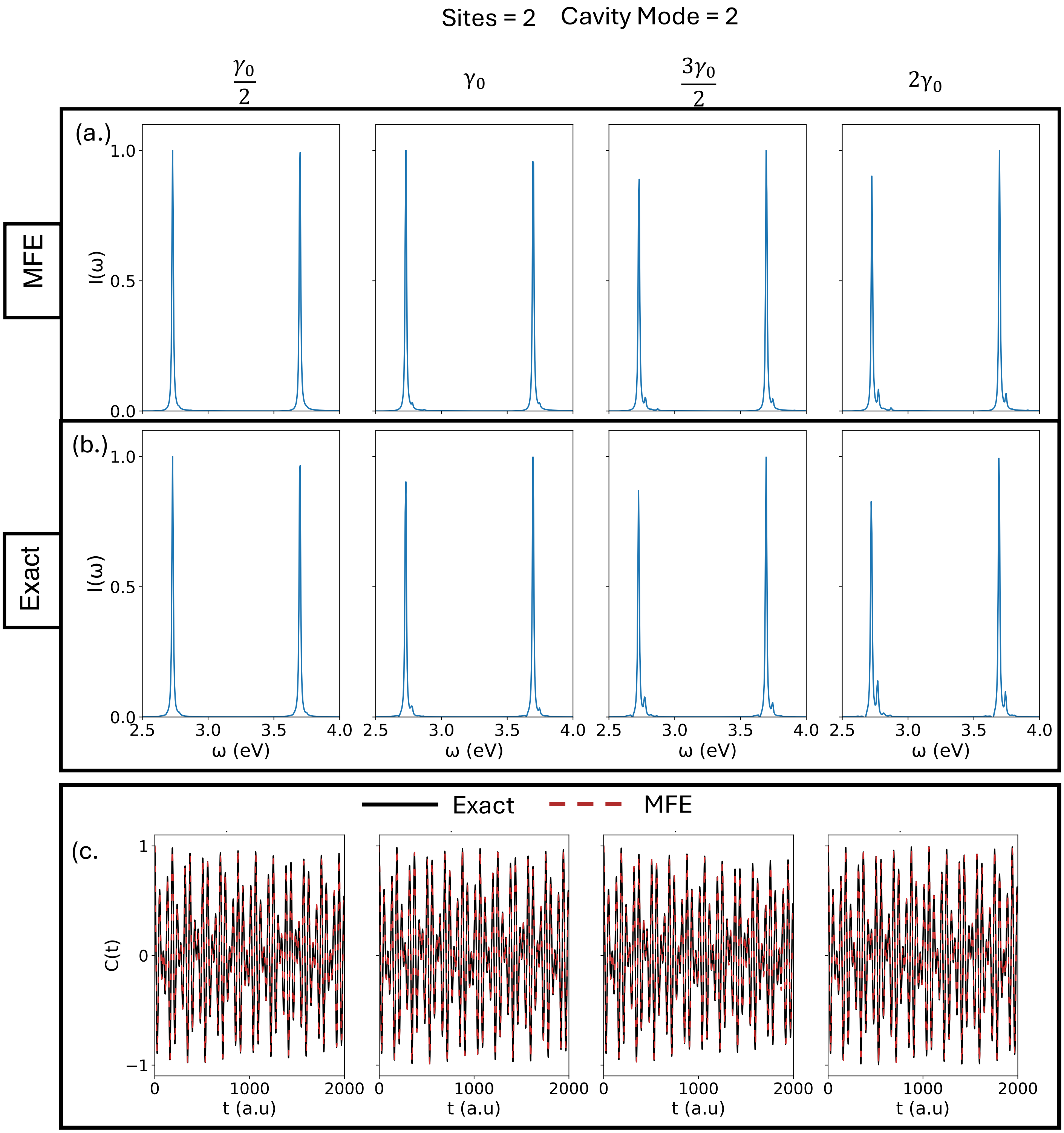

Figure S4: Polariton spectra with $N = 2$ calculated using the mean field Ehrenfest (MFE) method (top row) and the exact diagonalization (bottom row) approach for different phonon coupling strengths, using a reference coupling constant of $\gamma_0 = 3.8898 \times 10^{-5}$ a.u. and a phonon frequency of $\omega = 400$ cm^{-1}.

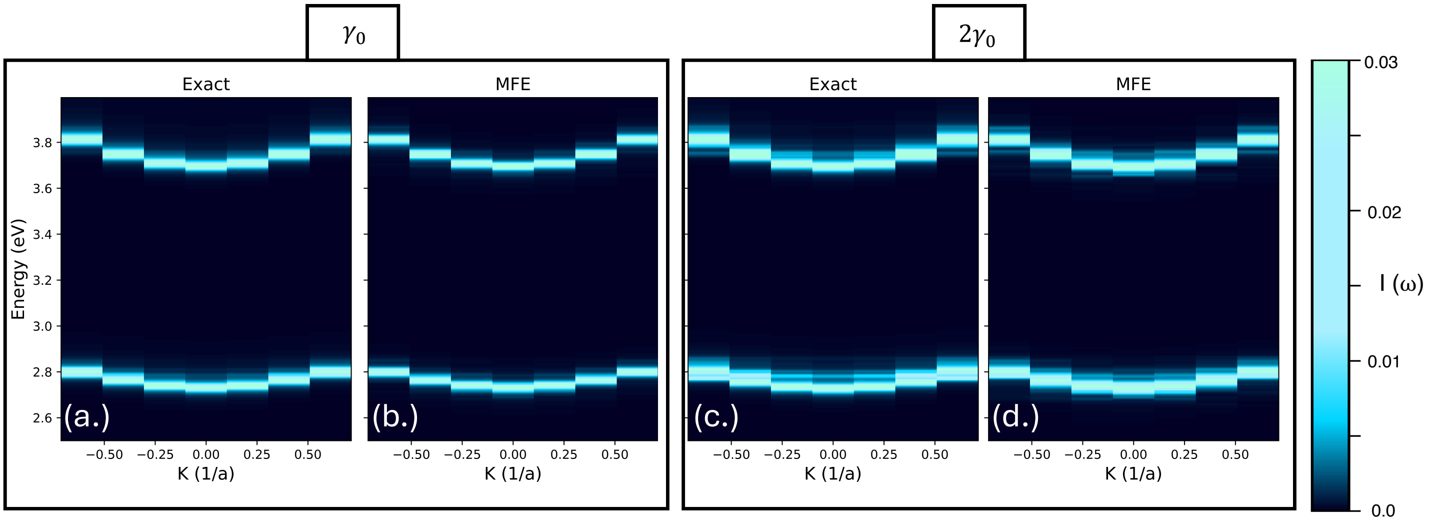

Figure S5: Exciton-polariton angle-resolved spectra calculated using Exact Quantum Dynamics (panels a and c) and the Mean Field Ehrenfest (MFE) method (panels b and d), for different phonon coupling strengths, using a reference coupling constant of $\gamma_0 = 3.8898 \times 10^{-5}$ a.u. and a phonon frequency of $\omega = 400$ cm^{-1}

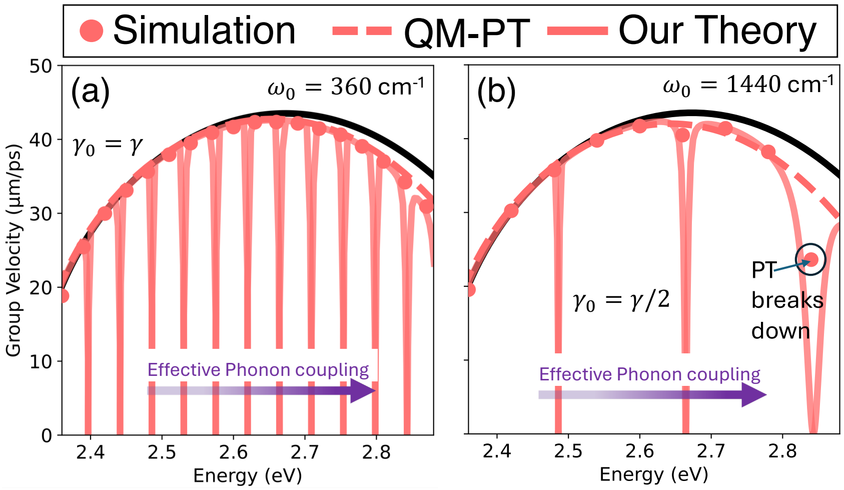

Figure S6: Comparison of group velocities obtained from MFE simulations (red circles) with two theoretical approaches: (1) a quantum mechanical perturbative approach [13] (QM-PT) (dashed line), and (2) the analytical theory (solid red line) presented in this work. (a) Low phonon frequency case with $\omega_0 = 360$ cm^{-1}, where the exciton-phonon coupling strength is $\gamma = 7.3 \times 10^{-5}$ a.u. and the number of excitonic sites is $N = 30001$. (b) High phonon frequency case with $\omega_0 = 1440$ cm^{-1}, using a coupling strength of $\gamma = 5.85 \times 10^{-4}$ a.u. and $N = 40001$ excitonic sites. Note that we have used low phonon coupling to ensure the accuracy of the QM-PT approach.

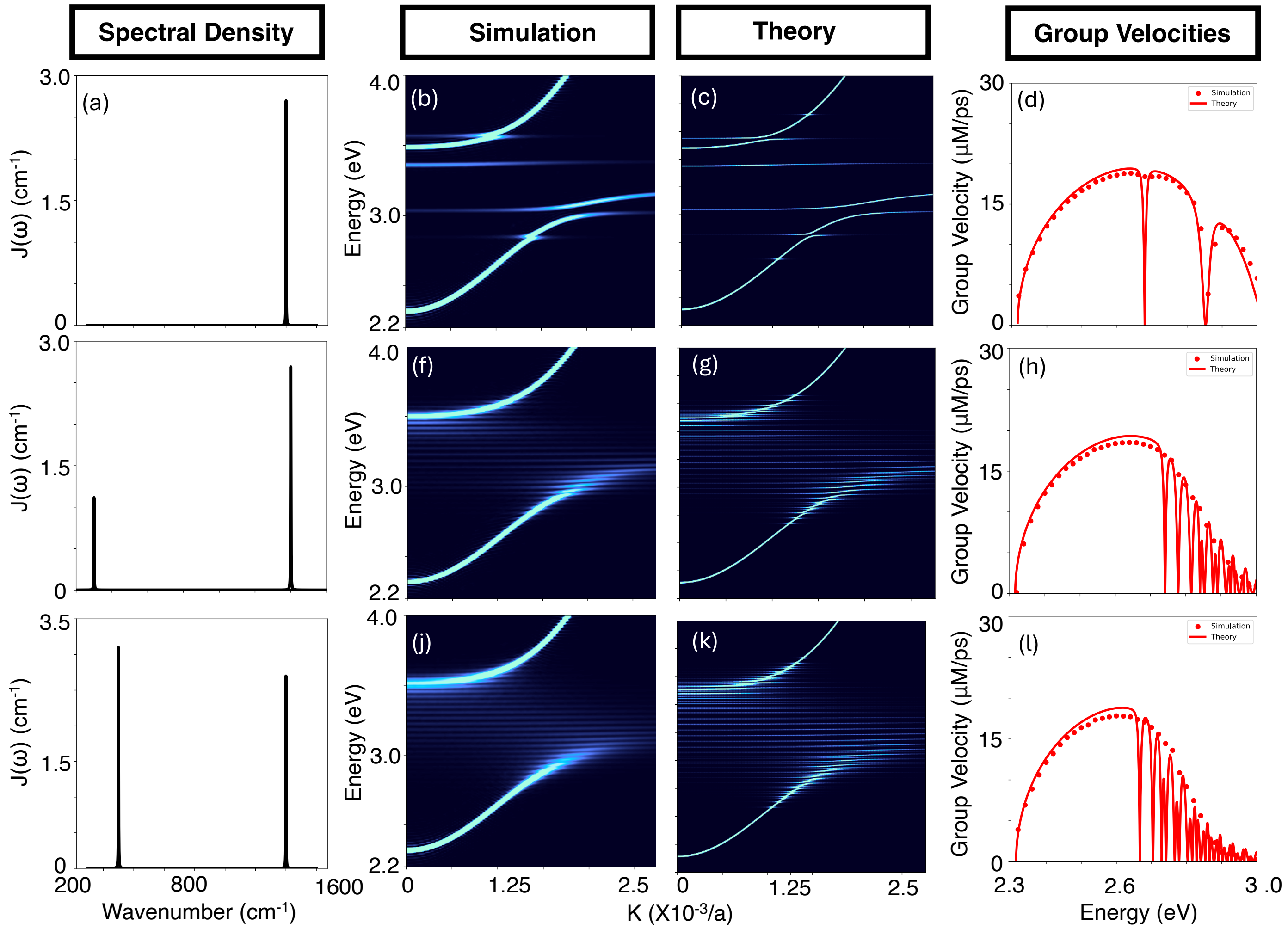

Figure S7: Comparison of angle-resolved spectra with Quantum Dynamics (MFE) simulations (b, f, j) and the proposed analytical theory (c, g, k) for various spectral densities (a, e, i) in two-mode systems with phonon frequencies $\omega_0 = 300$ cm^{-1} and $\omega_1 = 1400$ cm^{-1}. For the same systems, the corresponding group velocities (d, h, l) are compared between MFE results (red dots) and the analytical theory (solid red lines). The coupling strength of ω_0 is varied across rows: $\gamma_0 = 0$ a.u. (a–d), $\gamma_0 = 8.337 \times 10^{-5}$a.u. (e–h), and $\gamma_0 = 1.389 \times 10^{-4}$a.u. (i–l), while the coupling strength for ω_1 is fixed at $\gamma_1 = 2.8 \times 10^{-4}$ a.u. for all calculations.

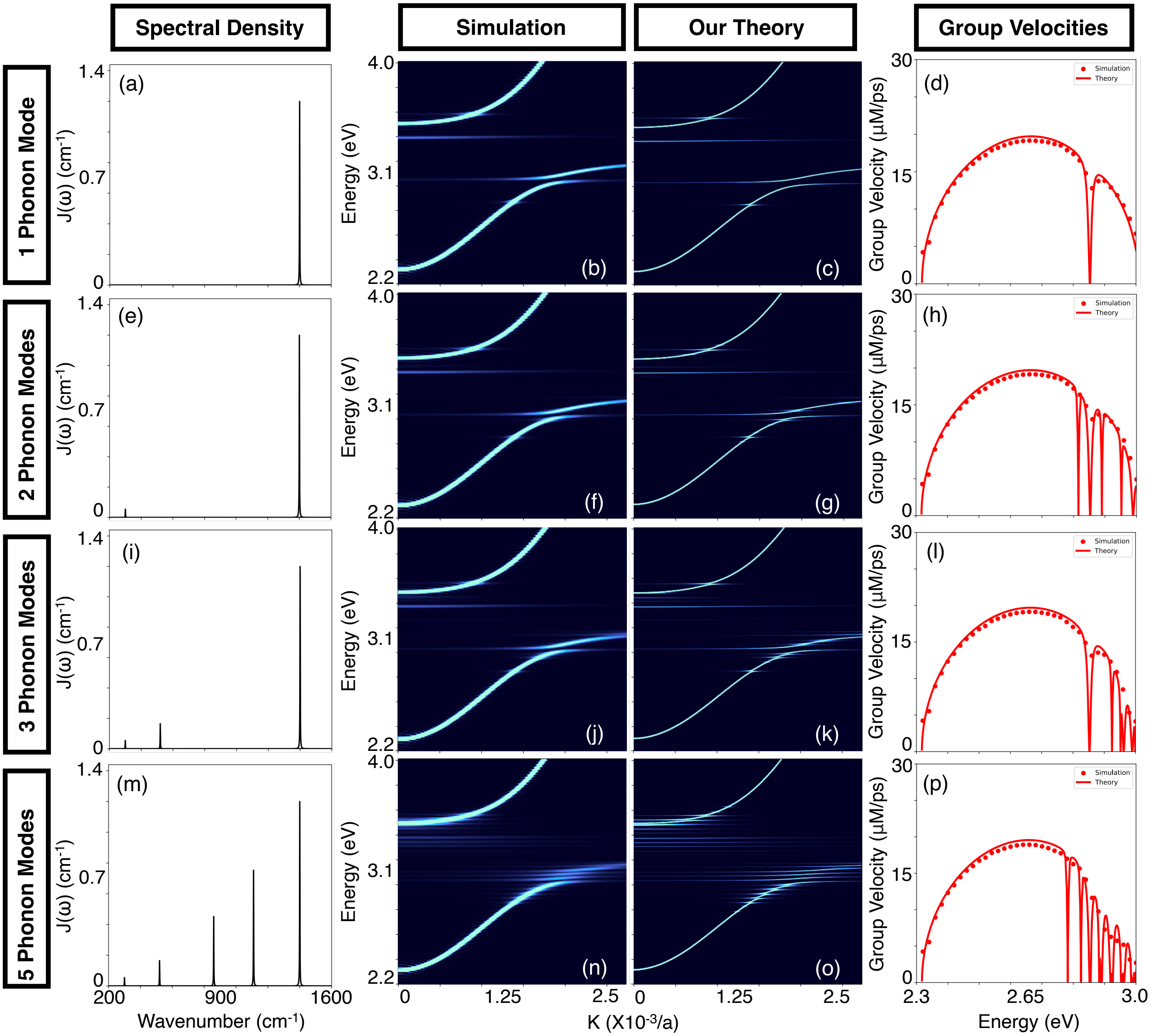

Figure S8: Comparison between Quantum Dynamics (MFE) simulation and proposed analytical theory including one (a-d), two (e-h), three (i-l), and five phonon modes (m-p), respectively. The leftmost column (a, e, i, m) presents the spectral densities of the considered phonon modes considered, being $\omega_0 = 300$ cm^{-1}, $\omega_1 = 520$ cm^{-1}, $\omega_2 = 860$ cm^{-1}, $\omega_3 = 1110$ cm^{-1}, $\omega_4 = 1400$ cm^{-1}, in ascending order. The central double column (b-c, f-g, i-k, n-o) is a side-by-side comparison of the simulated (left) and the analytical produced (right) polariton dispersion, increasing the number of phonon modes. The rightmost column presents the group velocities extracted from direct simulation (red dots) and the analytical theory (red solid lines). For this simulation the coupling constants used are $\gamma_0 = 1.85 \times 10{-}5$ a.u , $\gamma_1 = 4.23 \times 10{-}5$ a.u , $\gamma_2 = 8.99 \times 10{-}5$ a.u , $\gamma_3 = 1.32 \times 10{-}4$ a.u , $\gamma_4 = 1.86 \times 10{-}4$ a.u.

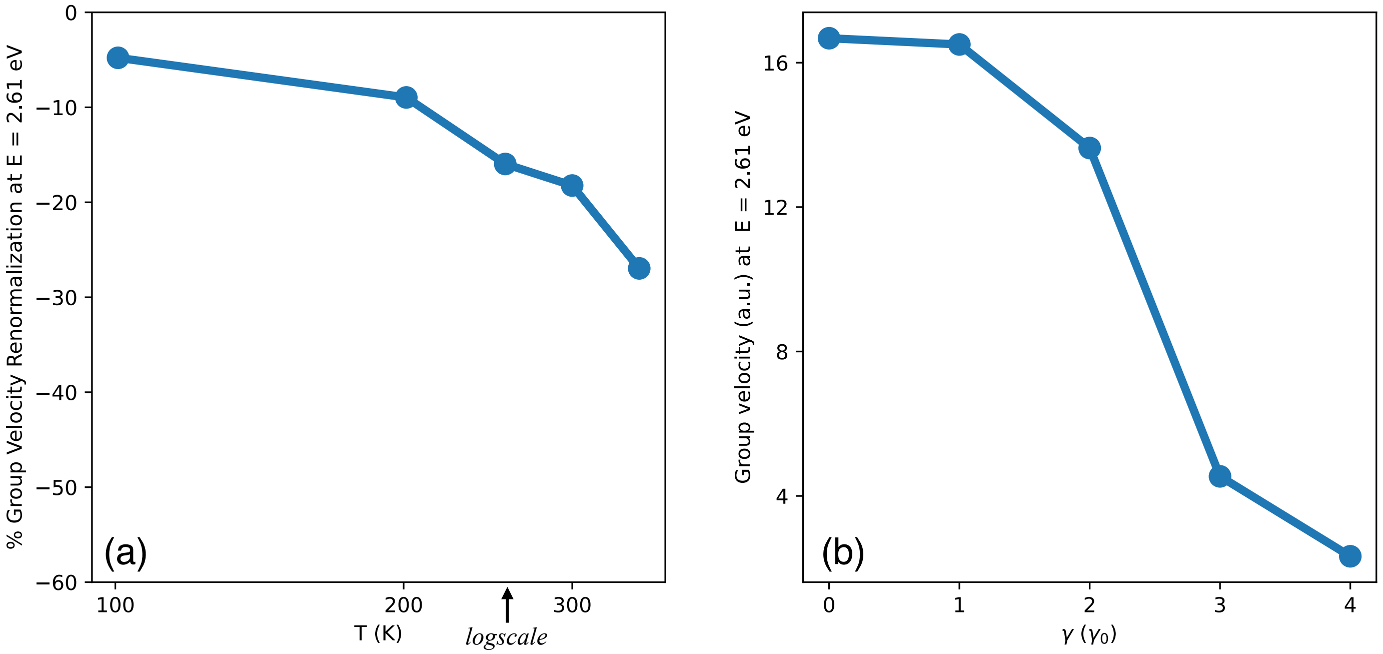

Figure S9: (a) Percent Group Velocity renormalization at various temperatures (compared to $\gamma = 0$) for $\omega = 360$ cm^{-1}, $\gamma = 2.92 \times 10^{-4}$ a.u, for a polariton initialized at $E = 2.61$ eV, at various temperatures between 100-350K. (b) Group velocity at differing amounts of phonon coupling for $T = 300$K, where $\gamma_0 = 1.46 \times 10^{-4}$ a.u, where all other parameter are identical to that in (a), for comparison.

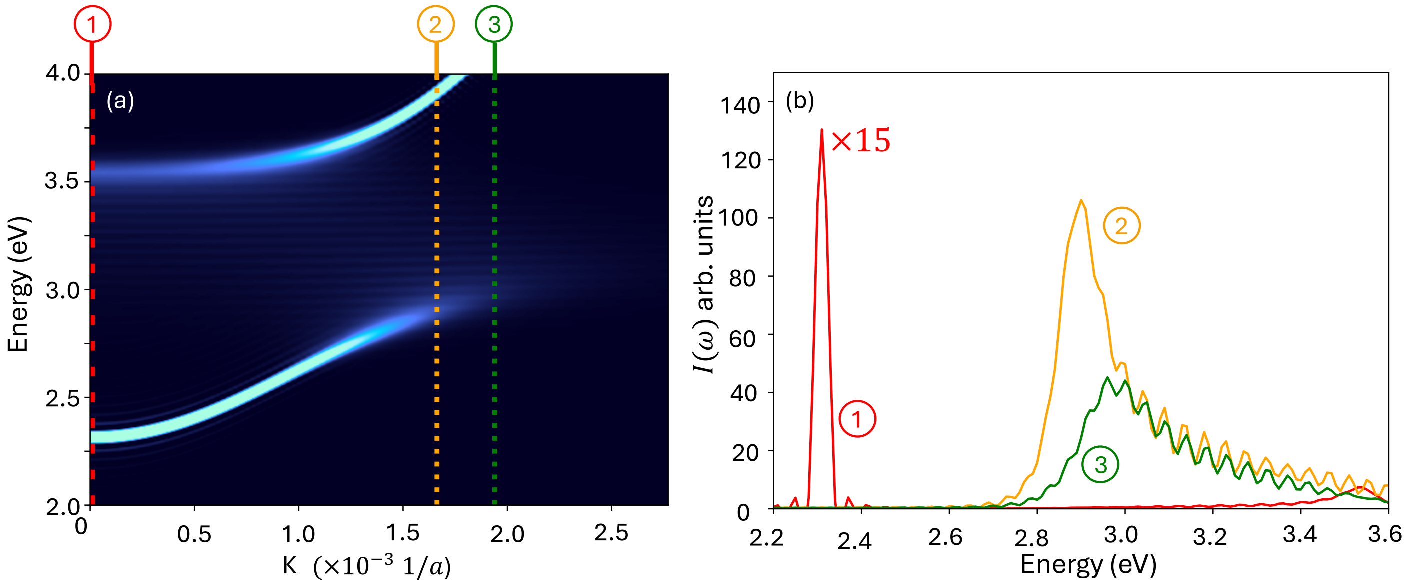

Figure S10: (a) Angle-resolved spectra obtained from MFE simulations for a low-frequency phonon case with $\omega_0 = 360$ cm^{-1}, exciton-phonon coupling strength $\gamma = 7.3 \times 10^{-5}$ a.u., and a chain length of $N = 30001$. Spectra are shown for three wavevectors k.

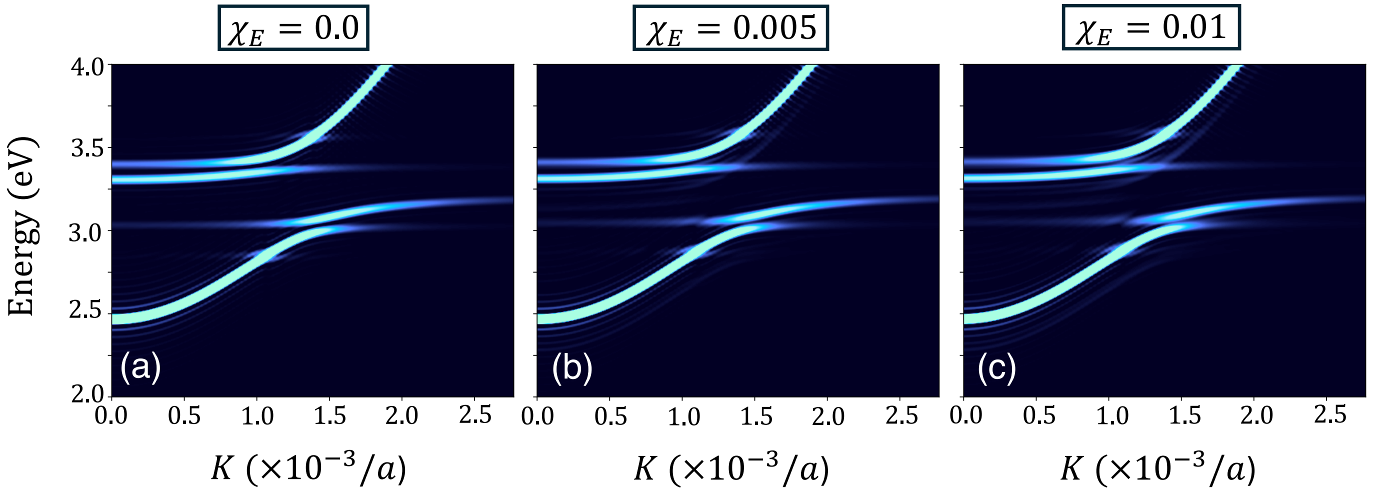

Figure S11: (a-c) Angle-resolved spectra obtained from MFE simulations of a single mode per site high-frequency mode $\omega_0 = 1440\ \mathrm{cm}^{-1}$), exciton-phonon coupling strength $\gamma = 2.923 \times 10^{-4}$ a.u., for $N = 40001$ at varying anharmonicity. (a) Presents the harmonic approximation of the polaron-polariton dispersion ($\chi_E = 0$). (b) Presents the polaron-polariton dispersion considering a mild anharmonicity ($\chi_E = 0.005$). Lastly, (c) presents the polaron-polariton dispersion considering a realistic anharmonicity for organic polymers ($\chi_E = 0.01$).

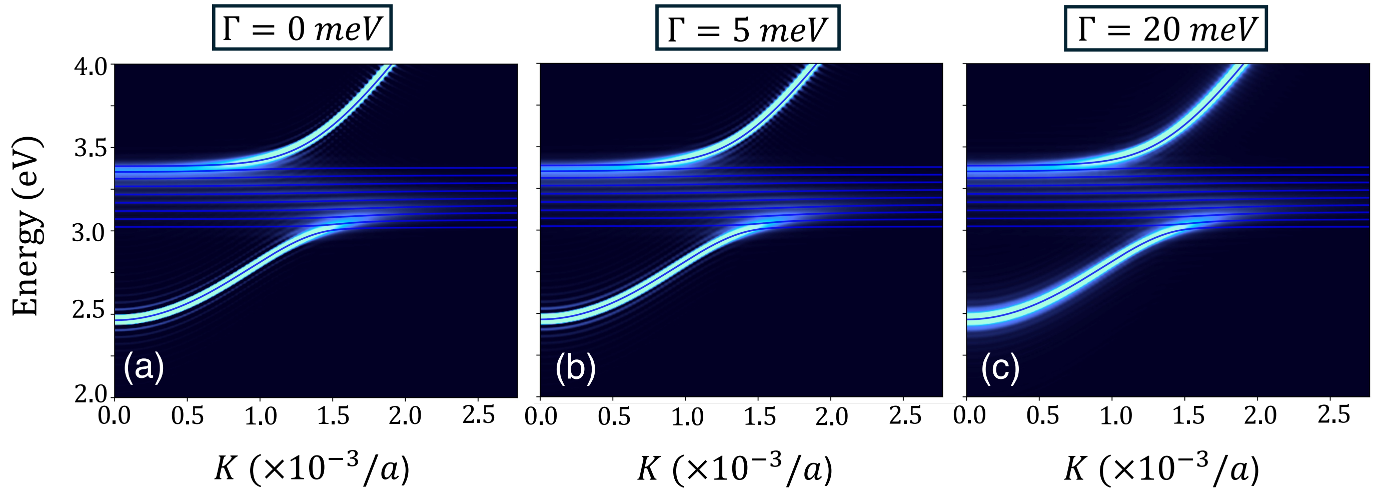

Figure S12: (a-c) Angle-resolved spectra obtained from MFE simulations of a single mode per site low-frequency mode $\omega_0 = 360\ \mathrm{cm}^{-1}$), exciton-phonon coupling strength $\gamma = 1.461 \times 10^{-4}$ a.u., for $N = 40001$ at varying cavity loss. (a) Presents the lossless cavity of the polaron-polariton dispersion ($\Gamma = 0$). (b) Presents the polaron-polariton dispersion considering a reasonable cavity loss ($\Gamma = 5$ meV). Lastly, (c) presents the polaron-polariton dispersion considering a large amount of cavity loss ($\Gamma = 20$ meV) to clearly demonstrate the line-broadening effect.

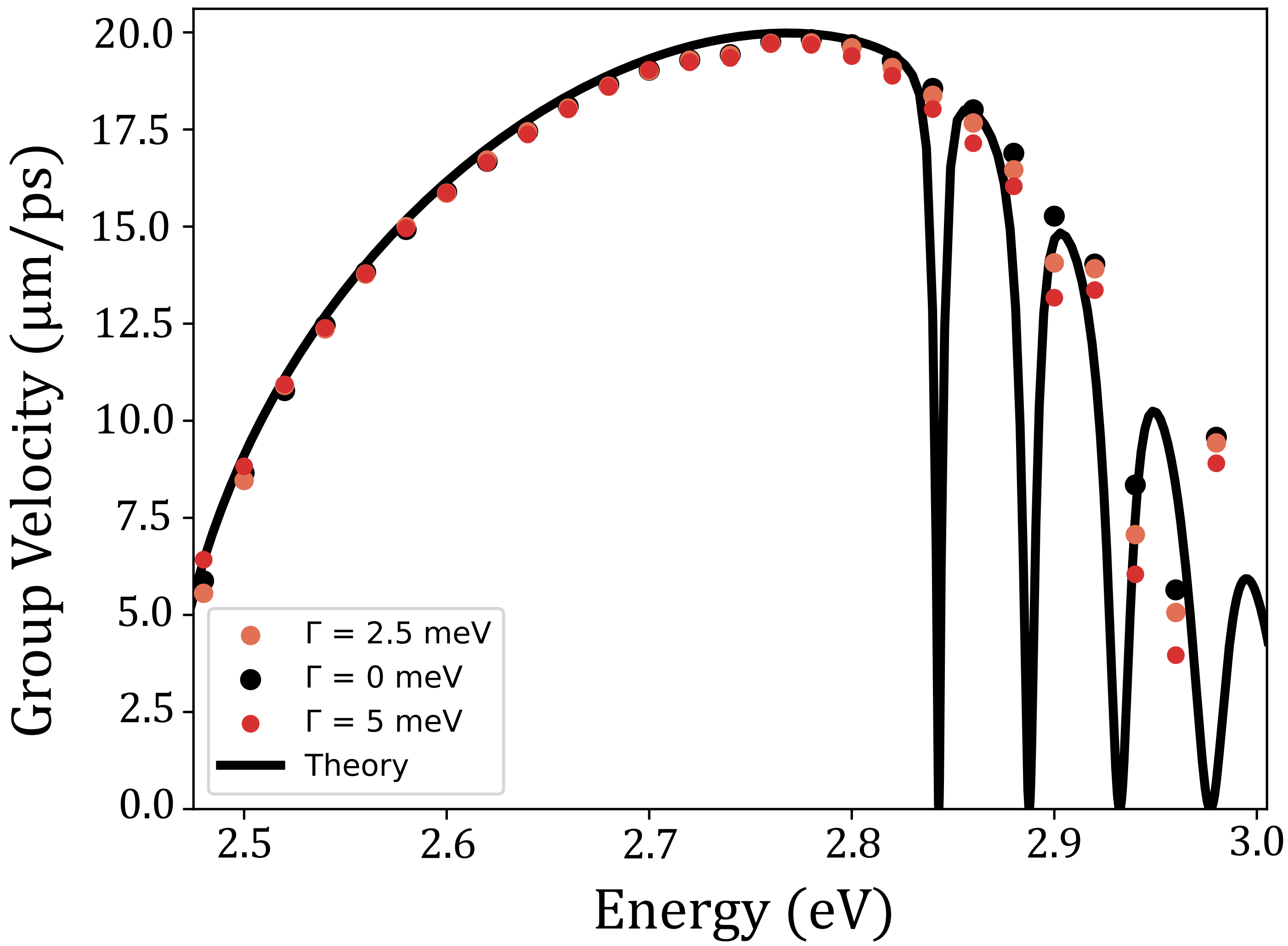

Figure S13: Group velocity obtained from MFE simulations at various cavity loss parameter Γ compared to the analytical theory, with $\omega_0 = 360$ cm^{-1}), exciton-phonon coupling strength $\gamma = 1.461 \times 10^{-4}$ a.u., for $N = 40001$. The black line represents the analytical theory (not loss dependent), where the circles represent MFE propagation at $\Gamma = 0$ (black), $\Gamma = 2.5$ meV (orange), $\Gamma = 5$ meV (red).

References

[1] Mandal, A. et al. Microscopic theory of multimode polariton dispersion in multilayered materials. Nano Letters 23, 4082–4089 (2023).

[2] Dmytruk, O. & Schiró, M. Gauge fixing for strongly correlated electrons coupled to quantum light. Physical Review B 103, 075131 (2021).

[3] Mandal, A. et al. Theoretical advances in polariton chemistry and molecular cavity quantum electrodynamics. chemrxiv-2022-g9lr7 (2022).

[4] Akimov, A. V. & Prezhdo, O. V. The pyxaid program for non-adiabatic molecular dynamics in condensed matter systems. Journal of chemical theory and computation 9, 4959–4972 (2013).

[5] Wang, L., Beljonne, D., Chen, L. & Shi, Q. Mixed quantum-classical simulations of charge transport in organic materials: Numerical benchmark of the su-schrieffer-heeger model. The Journal of chemical physics 134 (2011).

[6] Yamijala, S. S. & Huo, P. Direct nonadiabatic simulations of the photoinduced charge transfer dynamics. The Journal of Physical Chemistry A 125, 628–635 (2021).

[7] Ng, N. & Kolodrubetz, M. Many-body localization in the presence of a central qudit. Phys. Rev. Lett. 122, 240402 (2019).

[8] Koshkaki, S. R. & Kolodrubetz, M. H. Inverted many-body mobility edge in a central qudit problem. Phys. Rev. B 105, L060303 (2022).

[9] Taylor, M., Mandal, A. & Huo, P. Light-matter interaction hamiltonians in cavity quantum electrodynamics. Chemical Physics Reviews (2025).

[10] Shirley, J. H. Solution of the schrödinger equation with a hamiltonian periodic in time. Physical Review 138, B979 (1965).

[11] Oka, T. & Kitamura, S. Floquet engineering of quantum materials. Annual Review of Condensed Matter Physics 10, 387–408 (2019).

[12] Nguyen, H., Mandal, A., Mahajan, A. & Reichman, D. R. Mixed quantum-classical methods for polaron spectral functions. The Journal of Chemical Physics 163, 114105 (2025).

[13] Ying, W., Chng, B. X., Delor, M. & Huo, P. Microscopic theory of polariton group velocity renormalization. Nature Communications 16, 6950 (2025).

[14] Hong, Y., Xu, D. & Delor, M. Exciton delocalization suppresses polariton scattering. Chem 102759 (2025).

[15] Polak, D. et al. Manipulating molecules with strong coupling: harvesting triplet excitons in organic exciton microcavities. Chemical science 11, 343–354 (2020).

[16] Li, D. et al. Hybridized exciton-photon-phonon states in a transition metal dichalcogenide van der waals heterostructure microcavity. Physical Review Letters 128, 087401 (2022).

[17] Amano, M., Otsuka, K., Fujihara, T., Kondo, H. & Bando, K. Slow-light dispersion of a cavity polariton in an organic crystal microcavity. Applied Physics Letters 120 (2022).

[18] Kéna-Cohen, S. & Forrest, S. Room-temperature polariton lasing in an organic single-crystal microcavity. Nature Photonics 4, 371–375 (2010).

[19] Mackie, C. J. et al. The anharmonic quartic force field infrared spectra of hydrogenated and methylated pahs. Phys. Chem. Chem. Phys. 20, 1189–1197 (2018).

[20] Jasrasaria, D., Mandal, A., Reichman, D. R. & Berkelbach, T. C. Simulating anharmonic vibrational polaritons beyond the long wavelength approximation. The Journal of Chemical Physics 162, 014109 (2025).

[21] Spohn, H. Kinetic equations from hamiltonian dynamics: Markovian limits. Reviews of Modern Physics 52, 569 (1980).

[22] Balasubrahmaniyam, M. et al. From enhanced diffusion to ultrafast ballistic motion of hybrid light–matter excitations. Nature Materials 22, 338–344 (2023).

[23] Tichauer, R. H., Sokolovskii, I. & Groenhof, G. Tuning the coherent propagation of organic exciton-polaritons through the cavity q-factor. Advanced Science 10, 2302650 (2023).
